# Regulatory landscape enrichment analysis (RLEA): a computational toolkit for non-coding variant enrichment and cell type prioritization

**DOI:** 10.1186/s12859-024-05794-7

**Published:** 2024-05-07

**Authors:** Samuel Rosean, Eric A. Sosa, Dónal O’Shea, Srilakshmi M. Raj, Cathal Seoighe, John M. Greally

**Affiliations:** 1 Department of Systems and Computational Biology, Albert Einstein College of Medicine, Bronx, NY 10461 USA; 2 Department of Genetics, Albert Einstein College of Medicine, Bronx, NY 10461 USA; 3 School of Mathematics, Statistics & Applied Mathematics, National University of Ireland Galway, Galway, H91 TK33 Ireland

**Keywords:** Cell-type prioritization, Non-coding enrichment, Chromatin architecture, GWAS

## Abstract

**Background:**

As genomic studies continue to implicate non-coding sequences in disease, testing the roles of these variants requires insights into the cell type(s) in which they are likely to be mediating their effects. Prior methods for associating non-coding variants with cell types have involved approaches using linkage disequilibrium or ontological associations, incurring significant processing requirements. GaiaAssociation is a freely available, open-source software that enables thousands of genomic loci implicated in a phenotype to be tested for enrichment at regulatory loci of multiple cell types in minutes, permitting insights into the cell type(s) mediating the studied phenotype.

**Results:**

In this work, we present Regulatory Landscape Enrichment Analysis (RLEA) by GaiaAssociation and demonstrate its capability to test the enrichment of 12,133 variants across the *cis*-regulatory regions of 44 cell types. This analysis was completed in 134.0 ± 2.3 s, highlighting the efficient processing provided by GaiaAssociation. The intuitive interface requires only four inputs, offers a collection of customizable functions, and visualizes variant enrichment in cell-type regulatory regions through a heatmap matrix. GaiaAssociation is available on PyPi for download as a command line tool or Python package and the source code can also be installed from GitHub at https://github.com/GreallyLab/gaiaAssociation.

**Conclusions:**

GaiaAssociation is a novel package that provides an intuitive and efficient resource to understand the enrichment of non-coding variants across the *cis*-regulatory regions of different cells, empowering studies seeking to identify disease-mediating cell types.

**Supplementary Information:**

The online version contains supplementary material available at 10.1186/s12859-024-05794-7.

## Background

Despite the immense number of genome-wide association studies (GWAS) performed to study human diseases and traits, understanding how to progress from variant identification to the testing of their properties in vivo remains a difficult task, as the variants alone are not readily associated with the cell type(s) in which they may be preferentially exerting their effects. Most DNA sequence variants implicated in GWAS occur in the non-coding majority of the human genome, and are explored with functional genomic approaches to identify the subset of variants with effects on gene expression or transcriptional regulatory processes [1]. Non-coding variants have been demonstrated to alter transcriptional regulatory elements such as enhancers [2, 3] and to have effects on chromatin state [4], a potential mechanism for mediating the organismal phenotype. Functional non-coding variants are thus defined by their effects at regulatory loci, using assays such as those identifying open (non-nucleosomal) chromatin [5], patterns that are highly cell type-specific [6]. Studies have demonstrated significant enrichment of GWAS variants within open chromatin regions (OCRs), suggesting a transcriptional dysregulatory mechanism for non-coding variation [7]. Mechanistically, non-coding variants have been shown to contribute to disease risk through transcription factor (TF) binding dysregulation resulting in altered chromatin organization [8, 9]. Given the cell type specificity of the regulatory landscape, a variant may overlap and have effects on a regulatory locus such as an enhancer active in one cell type, but have no effects in any other cell type as the locus is not otherwise used for transcriptional regulatory purposes.

A challenge in following up on GWAS or other studies of non-coding variants implicated in a disease or phenotype is to identify the cell type(s) in which these variants are active. Our assumption is that disease-relevant functional variants are likely to be more frequent in regulatory regions that are active in the cell or tissue types mediating the condition. With the increasing availability of data from chromatin state assays such as ATAC-seq [10] that enable the identification of loci involved in cell type-specific gene regulation, we sought to develop a computationally-efficient way to use GWAS results to identify disease-mediating cell types [11–13].

### Limitations of current methodologies

There are now tens of thousands of GWAS in the NHGRI-EBI GWAS Catalog [14] and over 1,400 chromatin accessibility (ATAC-seq) results in the ATACdb database [15]. Tools are needed that can identify the tissue or cell types in which non-coding sequence variants are active [16–18], working with potentially very large sources of data. To investigate the relationship between variants and cell type effects, three approaches have been described. FUMA (Functional Mapping and Annotation) [19] serves to annotate and visualize GWAS results but is limited by its inability to incorporate user-customized annotation data for analysis and include tissue enrichment results as part of its output. Although FUMA accommodates positional expression quantitative trait loci (eQTL) and cell/tissue specific chromatin data, it relies on loci that have been functionally annotated and only indirectly links GWAS loci with specific cell or tissue types. Additionally, since it is a web-based platform, it does not support batch submissions and requires consistent server maintenance which limits the submission of tasks.

The Genomic Regulatory Elements and Gwas Overlap algoRithm (GREGOR) was developed to identify cell types and tissues implicated by GWAS studies [20]. This algorithm generates a list of candidate variants by filtering for those in strong linkage disequilibrium (LD) (*r*^2 ^> 0.7) with trait-associated index SNPs from whole genome sequencing [20]. GREGOR examines the overlap of potential causal SNPs with tissue OCRs and calculates the number of trait-associated loci at which either the index SNP or at least one of its LD proxies overlaps with a regulatory region [20]. Finally, GREGOR estimates the likelihood of the observed overlap of GWAS SNPs relative to expectation using a set of matched control variants (∼500 randomly selected SNPs that match the index SNP). Although GREGOR represents an advance in the study of the association of variants with the local regulatory landscape that defines cell types, its dependency on variants in LD introduces several complexities. GREGOR’s usage of LD variants can only be exclusively applied to human datasets, and is also limited to GWAS datasets where LD testing is relevant, excluding studies of other types of non-coding associations, such as de novo variant (DNV) association studies. In addition, GREGOR requires that the variant set and LD reference be derived from comparable genetic ancestries for biologically meaningful comparisons.

The third approach comparable to GaiaAssociation is the recently-described enrichment tool SpecVar, which uses both chromatin accessibility and gene expression data and conducts heritability enrichment analysis using GWAS statistics to identify phenotypically relevant tissues and cell types [21]. As the closest comparable approach, we focus our attention in this study on the comparison between the performance of GaiaAssociation and SpecVar.

Due to the growing availability of chromatin accessibility data in public repositories, along with an increased understanding of non-coding contributions to human phenotypes, the possibility of improving upon approaches like GREGOR or SpecVar has become timely. GREGOR’s LD approach is computationally costly, significantly reducing its performance capacity when compared to non-LD approaches. This efficiency cost is heightened as multiple studies and cell types are studied simultaneously. Its requirement of a pre-assembled LD reference library, and for variants and regulatory features to be aligned to the GRCh37 reference genome limit its applicability as chromatin accessibility technologies expand in their use.

To address these potential limitations and to test for the enrichment of loci in the regulatory elements of different cell types, we developed the GaiaAssociation package to perform Regulatory Landscape Enrichment Analysis (RLEA). Unlike previous enrichment software, GaiaAssociation evaluates the distribution of variants between the open chromatin architectures of cell types and does not require LD information, since all comparisons are made against their own null hypothesis. The implementation of RLEA partitions the genome into equally sized windows and then models the number of overlaps between single nucleotide variants (SNVs) or small indels and ATAC-seq peaks in each window as a binomial random variable. For the entire genome, the number of overlaps is modeled as a sum of independent binomial random variables, estimated using saddlepoint approximation [22]. GaiaAssociation allows users to efficiently test variant enrichment from multiple loci sets against the chromatin data of numerous cell-types simultaneously. This method highlights candidate cell types mediating the effects of genetic variants on the phenotype, a valuable post-GWAS insight, while also clustering cell types by their OCR profiles.

## Implementation

### Regulatory landscape enrichment analysis (RLEA): statistical design

Statistical testing by GaiaAssociation begins with creating a null distribution–a binomial distribution–for each GaiaAssociation window based on a specific local environment. This null distribution is built relative to the density of OCRs and number of loci in that GaiaAssociation window. In doing this we ensure a null distribution that does not naively assume that either a locus or an OCR could land anywhere in the genome, instead considering only the local environment where the loci was found. Taking every GaiaAssociation window that contains a locus, we examine the chance of this variant falling into an OCR (or not) based on the proportion of open chromatin within that GaiaAssociation window. If a particular locus falls within a GaiaAssociation window where no OCRs are found, there is a 0% chance of a loci landing in an OCR. The null distribution (and binomial test) for this GaiaAssociation window will reflect this point. In the example where a locus falls in a GaiaAssociation window with 98% OCR coverage, the null distribution for that GaiaAssociation window will reflect the high probability of overlapping with an OCR, giving a relative probability related to the local region where the locus was found. The final null distribution for p-value generation is calculated by summing the null distributions for all individual GaiaAssociation windows across the genome for a provided cell type. Thus, when the global overlap count of loci which landed in open chromatin regions is considered, the null distribution is constructed considering each GaiaAssociation window’s local environment.

### Implementation of regulatory landscape enrichment analysis (RLEA)

**Fig. 1:**
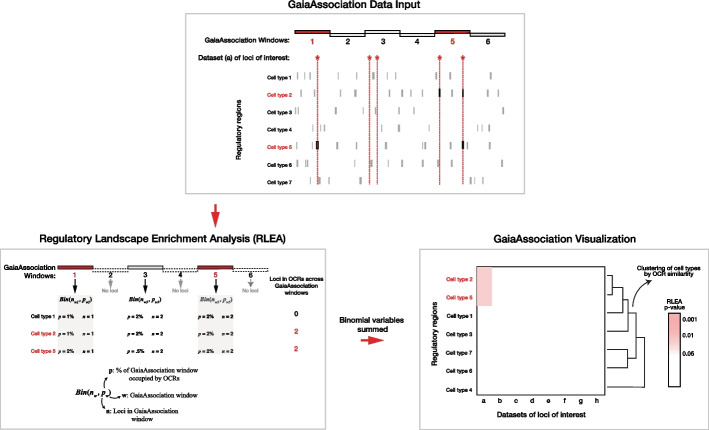
GaiaAssociation platform architecture. This flowchart demonstrates the data input, Regulatory Landscape Enrichment Analysis (RLEA), and visualization stages available through GaiaAssociation. During the data input stage, GaiaAssociation is provided a dataset of loci of interest (which could be GWAS loci or *de* novo variants, as examples, represented as red asterisks) along with cell type chromatin accessibility data (represented as narrow vertical rectangles). Each chromosome is divided into a set of GaiaAssociation windows (represented as broad horizontal rectangles) based on a user-defined window size. In the following step, loci of interest that land within GaiaAssociation windows (represented as red broad horizontal rectangles) are modeled as a binomial random variable for each cell type independently. During this RLEA stage, GaiaAssociation calculates the number of loci within a GaiaAssociation window (*n*) and the percentage of the GaiaAssociation window that is occupied by open chromatin regions (*p*) throughout the genome, modeling each as an independent binomial variable before summing these across all GaiaAssociation windows (*w*) by cell-type. This is then compared against the global overlap count between the loci data set and each cell type. This comparison provides a p-value representing the enrichment likelihood of a set of loci of interest within a cell type set against a random distribution null hypothesis. The final stage visualizes the enrichments calculated by GaiaAssociation as a heatmap matrix between all cell types and datasets of loci of interest, while clustering cell types by their shared OCR profiles

The architecture of RLEA by GaiaAssociation is represented in Fig. 1. To calculate the sum of independent but non-identical binomial random variables, RLEA takes a user-provided chromosome size file based on the genome build of choice and divides the chromosomes into GaiaAssociation windows of equal length, as close to a user-defined window size as possible (a default value of 100 kb is applied if no value is provided). For each GaiaAssociation window in which there is at least one query/SNP, a binomial random variable is modeled using the total number of loci which fall within that GaiaAssociation window, and the percentage of that window which overlaps a particular cell type’s OCRs, serving as the number of tests and the probability of success, respectively. The null distribution is then calculated as the sum of these independent binomial random variables. This summed distribution is then compared to the total genome-wide number of overlaps between a set of loci and a given OCR dataset. This comparison determines how likely it is to observe that number of overlaps considering the environment of each window independently. This comparison generates a p-value representing the likelihood of observing that many overlaps given a null hypothesis that each locus was randomly distributed within its local environment. This distribution is estimated using saddlepoint approximation [22], allowing the query set to be tested for enrichment, given the observed number of overlaps. This is summarized in the following formula below. β represents a binomial distribution, where β (*n*_*w*_*, p*_*w*_) is a binomial distribution defined by the variables (*n* & *p*). The binomial variable for GaiaAssociation window 1 (*w* = 1), would be defined by *n*_1_ and *p*_1_. The *n* signifies the number of trials, which here represents the number of loci within that GaiaAssociation window. The *p* signifies the probability of success, which in our usage case represents the proportion of the GaiaAssociation window covered by OCRs. This GaiaAssociation window (*w*) is therefore modeled as a single binomial distribution, and this distribution serves as the null distribution for that GaiaAssociation window. All overlaps (i.e. *overlaps*_*all*_, meaning the total number of overlaps across the entire genome) are then compared to the sum of all these null distributions, which are each an independent binomial distribution. These non-identical independent binomial variables are summed into a single null distribution to compare against for significance providing a p-value.

$${overlaps}_{all} ={\sum }_{w=1 }^{\# windows}\beta ({n}_{w}, {p}_{w})$$

For each GaiaAssociation window, the proportion that is occupied by OCRs is calculated for each cell type, and the total number of loci within that GaiaAssociation window is found. In the case of loci that overlap the OCR and have a length greater than one base pair, such as an insertion or deletion event, the size of each OCR in the GaiaAssociation window is extended to include the length of these extended loci. This is a slight adjustment to the more conventional single base pair loci calculation which is made to account for the possibility that an indel could land outside the OCR region but still overlap with it. This proportion of OCR coverage and the count of loci within that GaiaAssociation window, along with the total number of loci that overlap OCRs genome-wide, are used to calculate a p-value for global enrichment using the sinib package [22], which has been translated from R to Python and included with GaiaAssociation. This p-value is calculated for each cell type (*n* values) and loci dataset (*m* values) combination, to create an *n* x* m* matrix which describes the relative enrichment of these loci in each cell type.

### Interface for data input

The GaiaAssociation package requires four inputs for analysis: 1) a folder containing the chromatin regulatory region bed files of interest in a text (.txt) format, 2) a folder containing the genomic loci of interest in tab-separated values (.tsv) or comma-separated values (.csv) format, 3) a.csv file containing the chromosome sizes of the desired genome build, and 4) a folder location for the output of GaiaAssociation analyses.

### Data pre-processing

The most straightforward example of the use of GaiaAssociation is represented by the input of a list of loci to test for overlap with OCRs from multiple cell types. The package supports SNVs and small indels, not larger copy number variants, with the primary goal of supporting data typical of GWAS results. When entering multiple sets of genomic loci, the user can define cutoff values to filter for a minimum size requirement for a dataset, for example. We used a minimum cutoff of 200 genomic loci as a conservative threshold.

GaiaAssociation also permits the user to incorporate their own ATAC-seq loci or regulatory loci defined by other genome-wide assays (DNase-seq, ChIP-seq, etc.). The software tests the relatedness of the loaded regulatory profiles as a pre-processing step. Cell types with a greater degree of chromatin sharing (relatedness) cluster closely resulting in a smaller weight (closer to 0). Cell types with limited (or zero) chromatin relatedness receive a larger weight score (closer to 1) and cluster farther apart. GaiaAssociation provides a weight score (from 0–1) for all regulatory profiles, where degree of chromatin overlap between cell types is represented by smaller scores. This relationship between each pair of cell types is defined using the following formula:

$${Weight}_{ij} = 1 - (\frac{Overlap\, of\, i\, and\, j\, Peaks}{Sum\, of\, Cell\, Type\, i\, peaks})(\frac{Overlap\, of\, i\, and\, j\, Peaks}{Sum\, of\, Cell\, Type\, j\, peaks})$$

This weighted matrix is used to generate a dendrogram, based on the Euclidean distance metric and hierarchical clustering, using the SciPy8 algorithm [23], highlighting the similarities in chromatin organization between cell types.

### Interface features and functionality

GaiaAssociation also allows prioritization of regulatory regions that are cell type-specific. Accessible regions of chromatin ubiquitously shared across cell types possess a stronger signal compared to cell-type selective OCRs. However, unlike ubiquitous OCRs, cell-type specific OCRs harbor DNA sequence motifs corresponding to master regulators of cell identity and occur near selectively expressed genes [24]. For this reason, considering the degree of cell type OCR specificity can help clarify meaningful variant-cell type associations. Distinguishing global enrichment of loci in ubiquitous OCRs (near housekeeping genes for example) from cell-specific enrichment is a valuable feature for the study of non-coding variants. This degree of OCR cell type specificity can be examined via GaiaAssociation using the “peak uniqueness value” function. This function outputs the number of cell types that possess an overlapping OCR, allowing filtering prior to analysis as an option. If this function is provided with a value of 3, for example, then only OCRs found in three cell types or fewer are considered, removing OCRs shared by four or more cell types. This will allow for enrichment to be assessed on the chromatin regions unique to cell types, defined as cellular “fingerprints”. GaiaAssociation peak uniqueness results are highly specific and influenced by the number and class of cell types analyzed. Therefore the “peak uniqueness value” option is best utilized in highly discretionary circumstances across limited cell types within a single cell-type domain to avoid heavy imbalances created by the choice of input sets. This option was designed to increase the flexibility of GaiaAssociation to approach highly refined questions. Additional user-defined settings, such as GaiaAssociation window sizes and masks, are also available and described below.

Based on a given GaiaAssociation window size–defined by the user (with a 100 kb default size)–each chromosome is defined into GaiaAssociation windows of equal size. Due to the impossibility of dividing a number into ranges of precisely equal length, for example dividing a 305 kb chromosome into GaiaAssociation windows of 100 kb, a GaiaAssociation window size is determined using a mathematical ceiling process. In this process a chromosome is divided by the user provided GaiaAssociation window size, and the result of this division is rounded up to the nearest integer. In this example, 305 divided by 100 provides a value of 3.05, which when rounded using this ceiling process provides a value of 4. The chromosome is then divided into four GaiaAssociation windows, leaving a base window size of 76, which is calculated by rounding down 305/4. This process is done to provide a standardization for dealing with this remainder difficulty. In this case, dividing 305 kb by 4 still provides a remainder of 1. This remainder is distributed into each window 1 by 1 until no remainder is left. The result of dividing our 305 kb example is four windows of size 76, 76, 76, and 77 kb.

A user-defined mask allows for the subsetting of open chromatin data based on regions of interest. If, for example, a list of genes, enhancers, or promoters is provided, then only the OCRs that overlap these masked regions will be retained for analysis. For instance, provided a set of loci and genes related to a particular function/condition, GaiaAssociation can highlight cell types enriched for loci near these genes, and implicated in mediating the geneset’s function. The availability of these user-defined settings allows for the investigation of more precise questions via GaiaAssociation. The GaiaAssociation software source code for the execution of these functions has been provided in Additional file 1**.**

### Comparison of GaiaAssociation with GREGOR and SpecVar

GaiaAssociation and SpecVar / GREGOR comparisons were performed in accordance with the provided documentation provided for each software. The steps and choices made for our comparison are provided in Additional file 8.

## Results

### Examples of regulatory landscape enrichment analysis (RLEA) studies

We applied GaiaAssociation to identify the cell types through which variants may impact a trait or disease. We selected ATAC-seq data from 44 cell types, derived from the ATACdb database and from a published dataset of human brain samples [13]. The NHGRI-EBI GWAS catalog v1.0 was used to identify 412 studies containing a variety of traits that could potentially be mediated by one or more of the 44 selected cell types. All ATACdb datasets were converted to the current GRCh38.p13 genome build using the UCSC Genome Browser LiftOver function. Here we demonstrate how RLEA yields insights into the cell types potentially mediating the traits and diseases studied by GWAS.

**Fig. 2:**
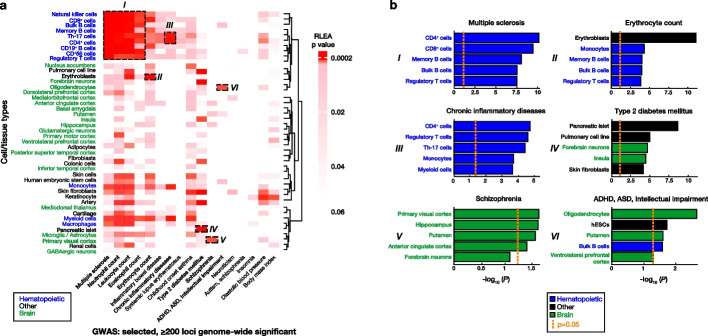
Regulatory Landscape Enrichment Analysis visualizes the enrichment of genomic loci across cell-type regulatory regions. We show the results of enrichment of significant loci from 17 GWAS and the *cis*-regulatory regions of 44 cell types, selected for their potential to mediate the GWAS phenotypes. **a** RLEA p-values were visualized using a matrix heatmap, using darker red to indicate the cell types that survive correction for multiple comparisons. We highlight a few associations in particular, (*I*) the association of multiple sclerosis with the regulatory loci of a number of immune cell types, (*II*) erythroblasts with erythrocyte count, (*III*) Th-17 and CD4 + lymphocytes with chronic inflammatory diseases (ankylosing spondylitis, ulcerative colitis, Crohn’s disease, psoriasis, sclerosing cholangitis), (*IV*) pancreatic islets with type 2 diabetes mellitus, (*V*) primary visual cortex with schizophrenia, and (*VI*) oligodendrocytes with attention deficit hyperactivity disorder (ADHD), autism spectrum disorder (ASD) and intellectual impairment. **b** The p-values for the top 5 cell type associations for each of the 6 highlighted examples from panel (a) are shown. The orange dashed lines at − log10 (*P*) = 1.30 represents a p-value cutoff of ≤ 0.05. These analyses illustrate some of the potential applications of RLEA

RLEA was applied to 17 of the 412 studies that contained ≥ 200 loci reaching genome-wide significance and could be mediated by the 44 selected cell types (Fig. 2a). A supplementary file has been provided to show these studies in greater detail [see Additional file 2]. Although non-exhaustive, our selection of cell types represents a diversity of functions ranging from neurologic, immunologic, metabolic, and hematological, highlighting how RLEA can be used in a hypothesis-free way if the researcher wants to examine a broad range of cell types. RLEA generated a heatmap matrix summarizing the significant and nonsignificant cell type-variant associations in red and white, respectively. Associations in darker red represent those that achieved multiple testing significance (*p*: ≤ 0.0002), while those in pink represent those with a *p* value ≤ 0.05. Cell types on the y-axis self-clustered into three groups: hematopoietic, brain, and other, with the latter representing broader functions. Bolded cell types were significantly enriched for variants from GWAS studies listed on the x-axis. RLEA highlighted nine studies (bolded on the x-axis) that were associated with six cell type classes (labeled *I -VI*).

The statistical association of these cell types and GWAS variants were analyzed as shown in Fig. 2b. Each panel summarizes the five cell types most significantly enriched for each GWAS study, with significance measured on the x-axis (− log_10_ *p* value). The orange dashed line indicates the significance threshold (*p*: ≤ 0.05). Results from panel *I* show that the cell types of the adaptive immune system (Regulatory T cells, CD4^+^, CD8^+^, and Bulk/Memory B cells) were significantly enriched for variants from multiple sclerosis, while variants from chronic inflammatory diseases were enriched in cell types of the adaptive and innate immune system (monocytes and myeloid cells) (panel *III*). Variants from studies of erythrocyte count were significantly enriched in erythroblasts (panel *II*, *p*: 7.22 × 10^–12^), while those from type 2 diabetes (T2D) mellitus were most enriched in pancreatic islets (panel *IV*, *p*: 2.51 × 10^–9^). Individuals with schizophrenia possessed variants enriched across a plethora of neuronal tissues with primary visual cortex having the strongest enrichment (panel *V*, *p*: 0.02). Variants from the study of ADHD, ASD, and intellectual impairment were enriched in the regulatory regions of a heterogenous group composed of neuronal, immune, and human embryonic stem cells (hESCs), with oligodendrocytes possessing the most significant enrichment (panel *VI*, *p*: 0.0002). These findings demonstrate how RLEA links GWAS results with cell types potentially mediating the studied trait or disease. These associations are consistent with prior studies linking GWAS variants with putative immune cell regulatory loci in autoimmune or inflammatory diseases [11, 12] or psychiatric disease and neurodivergent traits [25, 26]. Similarly, the enrichment of T2D variants further supports the genetic dysregulation of pancreatic islet cells as an underlying etiology in T2D [27], supporting the value of the RLEA approach.

To demonstrate the application of RLEA by GaiaAssociation beyond GWAS data, analysis was performed on 433 DNVs from individuals with epilepsy, of which 81 were non-coding. The epilepsy DNV data were acquired from the de novo mutation database [28] and analyzed to identify potentially mediating cell types or tissues. GaiaAssociation highlighted four brain regions and one neuronal cell type enriched for regulatory DNVs from individuals with epilepsy (Additional file 3 Fig. S1). Glutamatergic neurons (p: 0.038) was the only cell type enriched across epilepsy studies, consistent with its role in synaptic excitatory transmission that underlies hyperactivity and epileptogenesis upon dysregulation [29].

### Evaluation of GaiaAssociation performance

**Fig. 3:**
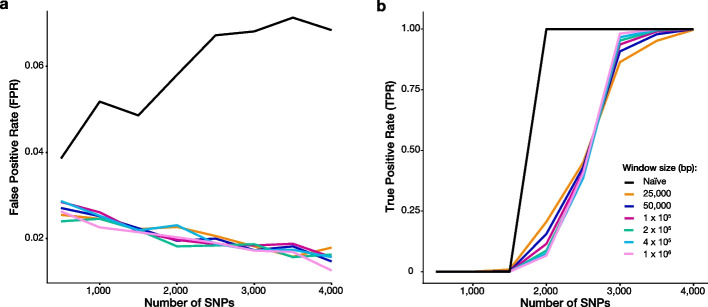
RLEA performance as a function of GaiaAssociation window size and loci count across 100,000 simulations. **a** False positive rate (FPR) declines as the number of SNPs increases and window size is defined. RLEA FPR was calculated for varying GaiaAssociation window sizes ranging from 25,000–1,000,000 base pairs in length. The naïve window size (black line) represents the entire genome in the absence of GaiaAssociation window selection. **b** Increased SNP number and GaiaAssociation window size are directly correlated with true positive rate (TPR) in RLEA (ignoring the naïve window). The TPR of the naïve window (black line) plateaus before all defined window sizes. Larger GaiaAssociation window sizes provided a higher TPR at 3,000 SNPs since decreased window sizes are associated with an increasingly conservative enrichment. All SNPs in this simulation analysis were selected at random from the UKB database and tested for enrichment in glutamatergic ATAC-seq peaks

Simulations were conducted to assess the true (TPR) and false positive rate (FPR) of RLEA as a function of SNP count and GaiaAssociation window size (Fig. 3). In the null simulations, SNPs were randomly selected from the genotyped and imputed SNPs in the UKB database. The association of these SNPs with regions defined by ATAC-seq peaks in glutamatergic neurons was assessed at a variety of window sizes, along with the “naive” binomial test, which can be regarded as using a window size incorporating the whole genome. All simulations were calculated for varying GaiaAssociation window sizes ranging from 25,000 to 1,000,000 bp in length. Null simulations demonstrate that the RLEA FPR declines as a GaiaAssociation window is defined and as the number of SNPs increases (Fig. 3a). Of note, the FPR increases for the naive binomial test as the number of SNPs used to assess association with the ATAC-seq peaks is increased. This suggests a weak colocalization of all SNPs with regions defined by ATAC-seq peaks, for which the naive binomial test fails to account. The FPR for RLEA decreases as the number of SNPs increases as the testing unit. Using a GaiaAssociation window–instead of the whole genome (naïve window)–accounts for the baseline colocalization of SNPs with ATAC-seq peaks explaining why the FPR does not increase as SNPs increase in simulations with a defined window. FPR analyses for RLEA demonstrate that the presence of a GaiaAssociation window provides an increasingly rigorous analysis and that the specificity of RLEA increases in studies with more SNPs.

Power simulations also drew SNPs from the UKB dataset to test for their association with the same ATAC-seq peaks as above. Fixed numbers of SNPs were drawn from a list that overlapped with ATAC-seq peaks and a list that did not. Increased SNP number and GaiaAssociation window size were directly correlated with TPR (Fig. 3b). The proportions of overlapping to non-overlapping SNPs correspond to enrichment levels of 30–50% over the expected value of the naive binomial distribution in the absence of any association (Additional file 4 Fig. S2). Unsurprisingly, the naive binomial has an advantage over RLEA as it does not account for the unsimulated association between SNPs and ATAC-seq peaks. This advantage is most obvious when the enrichment and number of SNPs are low. At moderate to higher values for enrichment and SNP numbers, the power of RLEA and the naive binomial are equivalent, with RLEA having the advantage of a lower FPR. The choice of window size, except for the genome-wide window in the naive binomial, will only lead to significantly different results if the proportion of the window occupied by ATAC-seq peaks differs significantly between them. These simulations indicate that selective GaiaAssociation window sizes in RLEA are associated with increasingly stringent testing and sensitivity. Importantly, these simulations demonstrate that FPR and loci number are inversely correlated and suggest a minimum threshold of ≥ 200 loci. Although simulations suggest this threshold, GaiaAssociation can effectively analyze datasets with any loci count, as all null distributions are built relative to the density of OCRs and number of loci per GaiaAssociation window. The TPR and FPR simulations here should serve as a reference to determine the desired level of sensitivity and specificity.

Given that GWAS variants do not fully account for phenotype heritability, we examined how the selection for highly significant genome-wide loci influences cell type prioritization. Cell type prioritization by GaiaAssociation was compared between GWAS variants possessing a more stringent p-value of ≤ 5.0 × 10^– 8^ and a less stringent ≤ 1.0 × 10^– 5^ (Additional file 5 Fig. S3). Variants were acquired from the EMBL-EBI GWAS catalog and tested against the same 44 cell types analyzed in Fig. 2a. Studies highlighted in Fig. 2b (labeled Additional file 5 Fig. S3a-f) and three additional studies from Fig. 2a–systemic lupus erythematosus (Additional file 5 Fig. S3g), leukocyte count (Additional file 5 Fig. S3h), and inflammatory bowel disease (Additional file 5 Fig. S3i)–were used for this comparative analysis. Cell type prioritization between variants from both p-value groups demonstrated strong concordance and positive association (R^2^ ranges: 0.82–0.996). Multiple sclerosis (Additional file 5 Fig. S3a) exclusively received a lower correlation score (R^2^: 0.82) due to the limited number of loci available for enrichment testing after filtering for those with a p-value ≤ 5.0 × 10^–8^. The remaining GWAS studies (Additional file 5 Fig. S3b–i) exhibited a substantial correlation (R^2^: 0.92–0.996) between both variant groups evidencing that cell type prioritization by GaiaAssociation is negligibly influenced by SNP *p* value threshold. The linear diagonal trendline (dashed red line) indicates that the regression model provides an excellent fit, and that most cell types were ranked analogously between both variant groups. Notably, the most highly ranked cell type–receiving a score of 1 and labeled in each plot–was concordant between both *p* value groups across all nine GWAS studies.

### Cell type enrichment comparison between GaiaAssociation and GREGOR

We compared the performance of GaiaAssociation with GREGOR on the six GWAS studies highlighted in Fig. 2b. To use GREGOR’s LD assembly function, GWAS variants and ATAC-seq datasets were converted from GRCh38 to GRCh37 coordinates using the UCSC LiftOver function. Across the six GWAS studies, GREGOR detected a greater level of non-specific global enrichment (Additional file 6 Fig. S4). GaiaAssociation provided a more rigorous analysis of loci from the ADHD, ASD, and Intellectual impairment study, highlighting 6 significant cell types out of 44 (*p* ≤ 0.05) (Additional file 6 Fig. S4a). GREGOR provided significant enrichment scores for 43 out of the 44 cell types analyzed, emphasizing a major difference in the specificity of the results generated. In the schizophrenia GWAS, GaiaAssociation found significant enrichment for only 4 neuronal cell types while GREGOR highlighted 44 (Additional file 6 Fig. S4b). In studies of erythrocyte count and multiple sclerosis, both computational toolkits highlighted many cell types. GaiaAssociation and GREGOR highlighted 34 and 44 cell types in the erythrocyte count study, respectively, with erythroblasts having the strongest enrichment in both (Additional file 6 Fig. S4c). Concordantly, CD4^+^ and CD8^+^ cells were the most enriched immune cells for variants from MS across both programs (Additional file 6 Fig. S4d). In the study of T2D mellitus, both toolkits similarly found the largest variant enrichment in *cis*-regulatory regions of pancreatic islet cells, an established cell-type association. However, GREGOR also detected that all but one of the 44 cell types was significantly enriched for T2D variants. In the chronic-inflammatory disease GWAS set, GREGOR identified 32 significantly enriched cell types across the immune, neuronal, and “other” groups. GaiaAssociation only prioritized 15 cell types, 12 of which were from the immune system, concordant with our current understanding of inflammatory processes in chronic disease. The results from these six comparative analyses are provided in Additional file 7 Table S1.

While GREGOR could prioritize a likely mediating cell type consistently with GaiaAssociation, its results were overall extremely non-specific. Additionally, GREGOR’s need to include LD analysis, while valuable for expanding loci to population-level linked alleles, contributes a significant computational and memory burden demanding substantially longer computation times.

### Cell type enrichment comparison between GaiaAssociation and SpecVar

**Fig. 4:**
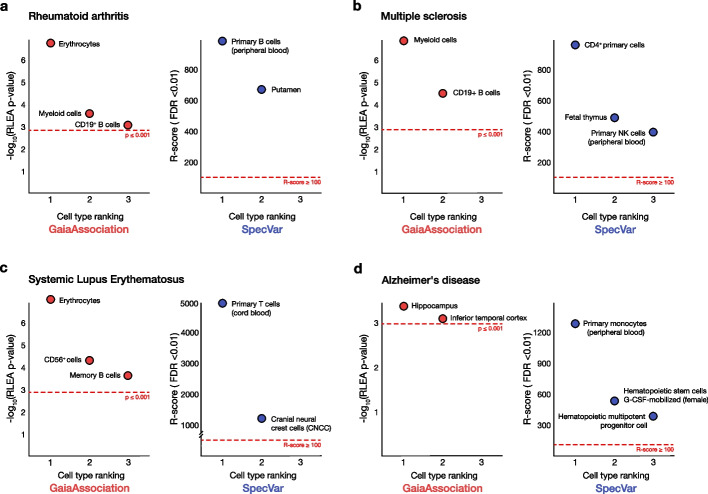
Comparative analysis of GaiaAssociation and SpecVar demonstrates comparable cell type prioritization. The three most enriched cell types achieving statistical significance across four GWAS phenotypes were highlighted for GaiaAssociation (in red) and SpecVar (in blue). **a** GaiaAssociation detected an enrichment of variants from individuals with rheumatoid arthritis in the regulatory regions of erythrocytes, myeloid cells, and CD19^+^ B cells. SpecVar similarly highlighted primary B cells and suggested the putamen region of the brain. **b** Multiple sclerosis variants are predicted by both algorithms to be enriched in regulatory loci of immune cell types and tissues. **c** In individuals with systemic lupus erythematosus (SLE), hoth algorithms identified immune cells but also included what are likely to be spurious associations, with GaiaAssociation highlighting erythrocytes and SpecVar implicating cranial neural crest cells. **d** In Alzheimer’s disease (AD), GaiaAssociation highlighted two brain regions, the hippocampus and inferior temporal cortex, while SpecVar predicted primary monocytes and blood stem cells (granulocyte-colony stimulating factor (G-CSF) mobilized hematopoietic stem cells and hematopoietic multipotent progenitor cells). An RLEA p ≤ 0.001 and an R-score ≥ 100 (FDR ≤ 0.01) served as significant enrichment criteria for GaiaAssociation and SpecVar, respectively

Comparative analyses were conducted to validate and assess the cell type enrichment highlighted through RLEA by GaiaAssociation. GaiaAssociation was compared to the recently described enrichment tool SpecVar, which synthesizes chromatin accessibility and gene expression data into regulatory categories and conducts heritability enrichment analysis with GWAS statistics to identify phenotypically relevant tissues and cell types [21]. A comparative analysis between GaiaAssociation and SpecVar was performed using four GWAS catalog phenotypes–rheumatoid arthritis (RA), multiple sclerosis (MS), systemic lupus erythematosus (SLE), and Alzheimer's disease (AD)–for which RLEA indicates mediating cell types (Fig. 4). All studies provided (1) summary statistics, (2) loci-specific effective sample sizes, (3) effect allele frequencies (EAF), and a (4) *p* value for each variant as required by SpecVar’s LDSC functionality [30]. Relevant context identification by SpecVar was performed on the full summary statistics for each dataset, while GaiaAssociation was executed on those SNPs that achieved a genome wide significance of *p* ≤ 5.0 × 10^–8^. The three most significantly enriched cell types (GaiaAssociation *p* value ≤ 0.001; SpecVar R-score ≥ 100 and FDR ≤ 0.01) across all phenotypes were used for comparison.

The results show that both GaiaAssociation and SpecVar have concordance in their cell/tissue type prioritizations, and each has what look to be implausible associations. B lymphocytes are predicted to be associated with rheumatoid arthritis by both approaches, with erythrocytes (GaiaAssociation) and putamen (SpecVar) likely to represent noise. Both techniques predict immune cells associated with multiple sclerosis and systemic lupus erythematosus, with brain tissues associated with Alzheimer’s disease by GaiaAssociation and blood cells by SpecVar. The approaches appear to be comparable in terms of generating reasonably plausible candidate cell types mediating the diseases studied by these GWAS studies.

There are some performance limitations to SpecVar that make it more difficult to implement. Unlike GaiaAssociation, SpecVar requires GWAS summary statistics, loci-specific effective sample sizes, effect allele frequencies, and variant p-values. Conversely, GaiaAssociation does not require as many data descriptors, uses minimal loci for comparable detection of potentially mediating cell types, and provides a beginner-friendly software interface. SpecVar is preloaded with 77 cell types constructed from paired expression and chromatin data taken from ENCODE and ROADMAP and is inflexible to the addition of further cell types not derived from these paired reference libraries, limiting the breadth of scientific inquiries that can be pursued. SpecVar has the advantage that it can leverage the heritability of variants, providing a useful alternative to GaiaAssociation when the number of loci achieving genome-wide significance is limited. This comparative analysis demonstrates that GaiaAssociation’s consideration of local environment comparably identifies phenotypically relevant cell types while requiring fewer genomic inputs and reducing computational barriers.

## Discussion

The integration of functional regions and regulatory loci using RLEA facilitates the translation of non-coding signals into biological and statistical insights that can be utilized in downstream research, such as drug target prioritization. Although approximately 90% of drugs fail during clinal trials, those supported by GWAS evidence are twice as likely to be approved for clinical use [31]. This high attrition rate emphasizes the need for computational strategies that incorporate novel genomic associations to define appropriate cell types in which the drugs should be active and therapeutic targets for the drugs [32]. Growing insight into functional non-coding regions that contribute to human disease will shape how molecular targets are prioritized. Recent studies have exemplified the value of integrating genome-scale approaches to prioritize potential drug targets to understand the therapeutic landscape across human disease [33, 34]. The strength of RLEA stems from its unique consideration of the local chromatin environment surrounding genomic loci, allowing investigators to analyze under-explored variants for enrichment across regulatory windows to inform decisions on cell-specific targets to pursue. RLEA can influence decisions regarding drug-target specificity by quantifying the degree to which a candidate variant affects one or many cell types simultaneously. Variants in regulatory regions shared by many cell types require careful consideration as their targeting may influence off-target cellular effects [35]. Maximizing the use of functional genomic information, RLEA quantifies the significance of genomic-variant associations to guide the selection of cell-specific drug targets, an invaluable contribution to drug prioritization and development.

The integration of non-coding variants and cell type data is a process that necessitates systematic and reproducible analytical approaches. The functional contributions of non-coding variants, especially near enhancer elements [36], DNase hypersensitivity regions [37], and chromatin marks [38], should be contextually considered. Collective observations indicate that non-coding variant effects are highly dependent on the genomic context and temporospatial activity of *cis*-regulatory elements [39–41], requiring computational toolkits that integrate context-specific chromatin data to identify regulatory variants that cause disease. Here we demonstrated how RLEA unbiasedly clustered 44 cell types according to their degree of shared regulatory regions. Cells from the adaptive immune system clustered tightly at the top (Fig. 2a; blue cell types) indicating their shared chromatin landscape and biological function. Notably, cells of the innate immune system (monocytes, myeloid cell, and macrophages) clustered separately from those of the adaptive system, highlighting differences in chromatin regulatory profiles between branches of the immune system. Brain cell types clustered together in the middle (Fig. 2a; green cell types), with microglia /astrocytes clustered closely to macrophages, consistent with microglia representing brain-resident macrophages [42]. Analysis of DNVs in individuals with epilepsy using GaiaAssociation highlighted brain structures and glutamatergic neurons, concordant with our current understanding of the pathogenesis of the condition. These examples highlight the clinical utility of RLEA and its ability to group cell types based on their chromatin accessibility reflecting cellular properties.

RLEA by GaiaAssociation presents two significant improvements over current computational models to address these limitations in the analysis of non-coding variants. Firstly, it does not utilize existing loci or gene annotations to determine cell-type associations, and instead relies on the chromatin architecture that defines cell types. Previous methodologies that directly associate variants with local genes by linkage disequilibrium or proximity, without considering chromatin context, can be misleading, especially for rare non-coding variants, where low allele frequencies and reduced power challenge our interpretation of mediating cell types [43]. Secondly, GaiaAssociation utilizes a local architecture dependent binomial test, utilizing the sinib method, which does not naively assume a null hypothesis where loci can be randomly distributed anywhere within a chromosome or genome. GaiaAssociation considers the local environment of each locus to make determinations on the probability of a loci landing in a cell-specific OCR within a given GaiaAssociation window. Through use of a definable GaiaAssociation window size, this method allows for fine grained considerations of local environments, which is paramount for studies including multiple cell types. Incorporating the regulatory landscapes of cell types is necessary for the study of non-coding variants and will be pivotal to realizing the clinical utility of whole genome sequencing data.

## Conclusions

In recent years there has been considerable need for the development of computational tools to guide the study of non-coding DNA sequence variation and cell type prioritization. In this work, we introduce RLEA by GaiaAssociation, a publicly available, comprehensive toolkit for the analysis of variant enrichment across cell-specific chromatin architecture. GaiaAssociation is user-friendly and requires minimal computational experience, increasing accessibility to the scientific community. It will reduce computational costs associated with large genomic studies, broadening the potential applications for this open-source software. Its flexibility ensures its malleability to highly particular research questions, based on a clearly defined null hypothesis and statistical method which is currently unavailable in alternative analytical methods. GaiaAssociation remains under active development and will continue to advance as we learn more about variant-cell type considerations.

### Supplementary Information

**Additional file 1:** GaiaAssociation source code. This python notebook file (.ipynb) includes the functional source code required to run GaiaAssociation.

**Additional file 2:** Description of Fig. 2 GWAS studies. This excel file (.xlsx) provides the EBI GWAS catalog title name, PMID, descriptive name (used in Fig. 2), sample sizes, and number of loci reaching genome-wide significance for all of the studies analyzed in Fig. 2.

**Additional file 3: Supplementary Figure 1.** GaiaAssociation reveals. De novo variant enrichment in regulatory regions of brain structures and glutamatergic neurons in individuals with epilepsy. De novo variants in individuals with epilepsy taken from the denovo-db database were analyzed using GaiaAssociation for cell type prioritization. The analysis consisted of 433 DNVs tested against 44 cell type regulatory profiles. GaiaAssociation highlighted four brain structures and one neuronal cell type. The orange dashed line at −log_10_ (*P*) = 1.30 represents a* p*-value cutoff of ≤ 0.05. These results highlight the applicability of GaiaAssociation to de novo variant data.

**Additional file 4:** **Supplementary Figure 2.** Analysis of the true positive rate (TPR) in GaiaAssociation across increasing SNP and enrichment levels. The TPR was tested using randomly genotyped and imputed SNPs from the UK Biobank database. These SNPs were overlapped against ATACseq data from glutamatergic neurons. Analyses were performed across seven GaiaAssociation window sizes. We studied a naïve GaiaAssociation window (entire genome) and GaiaAssociation windows ranging from to 25,000 – 1,000,000 bp. Each power analysis was conducted across enrichments of (**a**) 30%, (**b**) 35%, (**c**) 40%, (**d**) 45%, and (**e**) 50%. Enrichments are defined by the proportion of SNPs that overlap ATAC-seq regions (compared to those that do not); specifically the percentage over the expected value of the naive binomial distribution in the absence of any association. When the number of SNPs and enrichment are both low, the naive window size has an advantage as it does not account for the unsimulated association between SNPs and ATAC-seq peaks, explaining its higher FPR observed in Fig. 3a. At moderate to higher values of enrichment and SNP numbers, the power of RLEA and the naive binomial are equivalent, with RLEA having the advantage of a lower FPR. The TPR rate is achieved by all window sizes at lower SNP counts, and by all methods equivalently at higher enrichments

**Additional file 5**. **Supplementary Figure 3: Cell type prioritization by GaiaAssociation analysis is unaffected by the GWAS SNP p-value threshold**. Cell type prioritization by GaiaAssociation was compared between GWAS variants from the EMBL-EBI GWAS catalog possessing a p-value ≤ 5.0 x 10^−8^ (x-axis) and ≤ 1.0 x 10^−5^ (y-axis) across 44 cell type regulatory regions. Studies highlighted in **Figure 2b** (labelled **a-f)** and three additional studies from **Figure 2a** – systemic lupus erythematosus (**g**), leukocyte count (**h**), and inflammatory bowel disease (**i**) – were used for comparative analysis. Cell type prioritization between variants from both p-value groups demonstrated strong concordance and positive association (R^2^ ranges: 0.82 - 0.996) providing evidence that SNP selection by p-value threshold does not influence the selection of cell type. Cell types colored in red received an RLEA p-value ≤ 0.001 and achieved multiple testing significance with variants from both* p*-value thresholds. Cell types colored in pink and green received a RLEA* p*-value of ≤ 0.05 and > 0.05, respectively. The linear diagonal trendline (dashed red line) indicates that the 44 cell types were ranked analogously between both variant groups. Notably, the most highly ranked cell type (labelled in each plot) was concordant between both* p*-value groups across all nine GWAS studies.

**Additional file 6:** **Supplementary Figure 4.** Comparative enrichment analysis between RLEA by GaiaAssociation and GREGOR. GaiaAssociation performance was compared against GREGOR using ATAC-seq regions from 44 cell types and the GWAS studies highlighted in Fig. 2b. While there was a general concordance of enrichment for cell type rankings between the methods, GREGOR tended to show a non-specific enrichment in significance across most or all cell types, whereas GaiaAssociation was generally more selective, especially for the ADHD, ASD, and Intellectual impairment results in (**a**) and the schizophrenia results in (**b**).

**Additional file 7:** **Supplementary Table 1.** GaiaAssociation and GREGOR comparative analyses results.

**Additional file 8:** **Supplementary Methods 1.** Utilizing GREGOR and SpecVar for the purposes of comparisons with GaiaAssociation.

## Data Availability

GaiaAssociation is a free open-source software available to the scientific community. All data needed to reproduce the presented findings, including source code, package overview, and documentation manual are available at https://github.com/GreallyLab/gaiaAssociation and https://github.com/GreallyLab/gaiaAssociation-Example-Guide. The chromatin accessibility data that support the findings of this study are available from the ATAC-db v1.03 database at the following URL: https://bio.liclab.net/ATACdb/index.php. GWAS variants analyzed in the study are publicly available as open data from the NHGRI-EBI GWAS catalog: https://www.ebi.ac.uk/gwas/. The GaiaAssociation software source code and GWAS studies analyzed in Fig. 2 have been provided in Additional files 1 and 2, respectively. The steps required for comparisons between GaiaAssociation and GREGOR/SpecVar are available in Additional file 8 Methods 1. GaiaAssociation and GREGOR comparative analysis results have been provided in Additional file 7 Table S1.

## Abbreviations

RLEA: Regulatory landscape enrichment analysis
GWAS: Genome-wide association studies
DNA: Deoxyribonucleic acid
DNV: De novo variant
ATAC-seq: Assay for transposase-accessible chromatin with sequencing
eQTL: Expression quantitative trait loci
MS: Multiple sclerosis
RA: Rheumatoid arthritis
SLE: Systemic lupus erythematosus
AD: Alzheimer’s disease
GREGOR: Genomic regulatory elements and gwas overlap algorithm
SNP: Single nucleotide polymorphism
LD: Linkage disequilibrium
SNV: Single nucleotide variant
Indel: Insert-deletion
OCR: Open chromatin region
.txt: Text format
.tsv: Tab-separated values
.csv: Comma-separated values
DNase-seq: DNase I hypersensitive sites sequencing
ChIP-seq: Chromatin immunoprecipitation sequencing
T2D: Type 2 diabetes mellitus
ADHD: Attention-deficit hyperactivity disorder
ASD: Autism spectrum disorder
hESCs: Human embryonic stem cells
TPR: True positive rate
FPR: False positive rate
