# Supplementary material for: Regulatory landscape enrichment analysis (RLEA): a computational toolkit for non-coding variant enrichment and cell type prioritization
Source: BMC Bioinformatics. 2024 May 7;25:179. doi: 10.1186/s12859-024-05794-7 (PMC11075237; doi:10.1186/s12859-024-05794-7)
Supplement: Supplementary file 1 — Additional file 1: GaiaAssociation source code. This python notebook file (.ipynb) includes the functional source code required to run GaiaAssociation. [file 12859_2024_5794_MOESM1_ESM.pdf]

**Additional File 1: GaiaAssociation source code. This python notebook file (.ipynb) includes the functional source code required to run GaiaAssociation.**

```python
##import neccesary libraries
import pandas as pd
import pyranges as pr
import numpy as np
import matplotlib.pyplot as plt
from collections import defaultdict
import operator
import scipy
from scipy.cluster.hierarchy import dendrogram, leaves_list
import scipy.spatial.distance as ssd
import os, glob
import sys
import seaborn as sns
import statistics
import math
import argparse
from scipy.optimize import root
from scipy.stats import norm
import warnings


####

#### Gaia Association:

#### Compare chromatin accessibility data (e.g. ATAC-seq, DNase-seq) against loci sets (e.g. de-novo mutations, SNPs, rare variants) to detect cell-specific enrichment of loci.

### Named for James Lovelock's Gaia Hypothesis

####


##Primary function for associating ATAC and GWAS
def gaiaAssociation(atacLocation, gwasLocation, chromosomeSize, outputLocation, uniqueCount=0, lociCutoff=0, lociSelection = 0, subsettingRegion=0, windowSize = 100000):

    ## ensure all the given locations and files are accesible and real
    if not os.path.exists(atacLocation):
        sys.exit("Cell region folder cannot be found")
    if not os.path.exists(gwasLocation):
        sys.exit("Loci folder cannot be found")
    if not os.path.exists(chromosomeSize):
        sys.exit("Chrom size file cannot be found")
```

```
    if not os.path.exists(outputLocation):
        print("Output folder cannot be found, attempting to build it:")
        os.mkdir(outputLocation)
        if not os.path.exists(outputLocation):
            sys.exit("Output folder cannot be built")
        else:
            print("Output folder succesfully built:")

    ## pyranges is a slightly outdated method and receives warnings about not properly identifying true or false values in certain instances
    warnings.filterwarnings('ignore', module='pyranges')

    ##Bring in our atac files for each cell type
    dataFrameList = []
    cellNames = []
    for filename in glob.glob(atacLocation + "/" + '*.txt'):

        loopFrame = pd.read_csv(filename, sep="\t")

        ##this checks if there was no header, if the first column name is chr1, it shifts columns down into a row
        if "chr1" in loopFrame.columns[0] or "Chr1" in loopFrame.columns[0]:
            columnFrame = loopFrame.columns.to_frame().T
            loopFrame = pd.concat([columnFrame,loopFrame], ignore_index=True)

        ##Gives first three columns the names 'Chromosome', 'Start', "End"
        loopFrame = loopFrame.rename({loopFrame.columns[0] : 'Chromosome', loopFrame.columns[1] : 'Start', loopFrame.columns[2] : "End"}, axis=1)

        ##ensure start and stop are numeric values
        loopFrame["Start"] = pd.to_numeric(loopFrame["Start"])
        loopFrame["End"] = pd.to_numeric(loopFrame["End"])

        ##ensure the chromosome id doesnt have white space and is the string datatype
        loopFrame['Chromosome'] = loopFrame['Chromosome'].str.strip()
        loopFrame['Chromosome'] = loopFrame['Chromosome'].astype(str)

        ##add this dataframe to our list of atac-sets
        dataFrameList.append(loopFrame)

        ##Save cell type names by formatting the file names
```

```
        s = filename[:-4]
        cellNames.append(s[s.rindex('/')+1:])

    if(len(dataFrameList)==0):
        sys.exit("No Cell region files found")

    ##do some light formating to ensure all atac-seq have the same labeling
    for i in range(len(dataFrameList)):
        if "seqnames" in dataFrameList[i].columns:
            dataFrameList[i] = dataFrameList[i].rename(columns={"seqnames": "chr"})
        if "chr" in dataFrameList[i].columns:
            dataFrameList[i] = dataFrameList[i].rename(columns={"chr": "Chromosome"})
        if "start" in dataFrameList[i].columns:
            dataFrameList[i] = dataFrameList[i].rename(columns={"start": "Start"})
        if "end" in dataFrameList[i].columns:
            dataFrameList[i] = dataFrameList[i].rename(columns={"end": "End"})

        ##ensure there is a size column and a Size column, this redundancy exists because of different package dependencies request for formatting
        dataFrameList[i]["Size"] = dataFrameList[i]["End"] - dataFrameList[i]["Start"]

    ##create an empty matrix to store our associations in
    matrix1 = np.zeros((len(dataFrameList),len(dataFrameList)))

    print("Formatting Cell Regions: ")

    ##create list to store our pyRanges objects in, merge them to ensure regions arent double represented
    prRanges = []
    for i in range(len(dataFrameList)):

        ##this particular bit of circular coding creates a pyrange, merges itself to remove redundancy, turns it back into a dataframe, creates a size column, and then turns it back into a pyrange. This is because pyrange doesnt have a function to add a size column and merging removes the size column, this tedium is their fault, but it isn't super time intensive
        tempRange = pr.PyRanges(dataFrameList[i])
        loopRange = tempRange.merge(strand=False)
```

```
        df = pd.DataFrame(list(zip(loopRange.Chromosome, loopRange.Start, loopRange.End)),columns =['Chromosome', 'Start', 'End'])
        df["Size"] = df["End"] - df["Start"]
        testRange = pr.PyRanges(df)
        prRanges.append(testRange)

    if uniqueCount != 0:

        ## Create unique sets of ATAC based on a cutoff value of number of overlaps
        overlapCutoff = int(uniqueCount)

        ##create New locations to store unique regions
        prRangesUnique = []
        prFrames = []

        ##save the current dataframes to new location for altering (this may be redundant based on python memory handling)
        print("Finding Unique Cell Region Peaks Between Cell Types:")
        for j in range(len(prRanges)):
            prFrames.append(prRanges[j].df)

        ##Loop through each atac set and compare against all others, remove locations with overlaps greater than overlap cutoff
        for j in range(len(prFrames)):

            if( j != len(prFrames) - 1):
                print("Cell Type " + str(j+1), end = " - ")
                testFrame = pd.concat(prFrames[:j] + prFrames[j+1 :])
            else:
                print("Cell Type " + str(j+1), end = "\n")
                testFrame = pd.concat(prFrames[:j])



            allButMain = pr.PyRanges(testFrame)
            loopRange = prRanges[j].coverage(allButMain)
            mainFrame = loopRange.df
            mainFrame = mainFrame[mainFrame["NumberOverlaps"] <= overlapCutoff]
            mainFrame = mainFrame.drop(columns=['NumberOverlaps', 'FractionOverlaps'])
            mainRange = pr.PyRanges(mainFrame)
            prRangesUnique.append(mainRange)


```

```
            if not os.path.exists(outputLocation + '/unique_regions'):
                os.mkdir(outputLocation + '/unique_regions')
                if not os.path.exists(outputLocation + '/unique_regions'):
                    print("Output folder for new unique regions cannot be built")
                    uniqueSave = False
                else:
                    uniqueSave = True
            else:
                uniqueSave = True

            if uniqueSave == True:
                mainFrame.to_csv(outputLocation + '/unique_regions' + '/unique_regions_' + cellNames[j][:10] + "_" + str(uniqueCount) + '.txt',sep='\t')


        prRanges = prRangesUnique


    ## If a merging region file is included then subset our ATAC sets using this region set
    if subsettingRegion != 0:

        print("Subsetting Cell Region Peaks Based on given mask region file:")

        geneLocation = subsettingRegion
        geneFrame = pd.read_csv(geneLocation, sep="\t")

        geneFrame = geneFrame.rename(columns={"Chr": "Chromosome", "start": "Start", "end" : "End"})

        geneRange = pr.PyRanges(geneFrame)

        if "seqnames" in geneFrame.columns:
            geneFrame = geneFrame.rename(columns={"seqnames": "chr"})
        if "chr" in geneFrame.columns:
            geneFrame = geneFrame.rename(columns={"chr": "Chromosome"})
        if "start" in geneFrame.columns:
            geneFrame = geneFrame.rename(columns={"start": "Start"})
        if "end" in geneFrame.columns:
            geneFrame = geneFrame.rename(columns={"end": "End"})

        ## Only keep regions which intersect with this subsetting region
```

```
        overlappedForms = []
        overlappedRanges = []
        for item in prRanges:
            loopRange = geneRange.intersect(item)
            loopRange = loopRange.merge(strand=False)
            loopFrame = loopRange.df

            loopFrame["Size"] = loopFrame["End"] - loopFrame["Start"]

            loopRange = pr.PyRanges(loopFrame)

            overlappedForms.append(loopFrame)
            overlappedRanges.append(loopRange)

        prRanges = overlappedRanges
        dataFrameList = overlappedForms


    print("Comparing cell regions between cell types: ")

    ##this function loops through each cell type and compares it to every cell type with a lower index number than itself. the comparison is a simple overlap calculation, it then adds these values to its respective matrix
    for i in range(len(dataFrameList)):

        if( i != len(dataFrameList) - 1):
            print("Cell Type " + str(i+1), end = " - ")
        else:
            print("Cell Type " + str(i+1), end = "\n")

        for j in range(len(dataFrameList)):

            if i > j:

                loopRange = prRanges[i].intersect(prRanges[j])
                if loopRange.df.empty:
                    matrix1[i,j] = 1
                    matrix1[j,i] = 1
                else:
                    overlap = sum(loopRange.End) - sum(loopRange.Start)
                    sizeA = sum(prRanges[i].Size)
                    sizeB = sum(prRanges[j].Size)

                    weightValue = 1- ((overlap)/ (sizeA) * (overlap)/(sizeB))
```

```python

                    matrix1[i,j] = weightValue

                    matrix1[j,i] = weightValue

    ## If the user only included one atac-seq we need to skip steps which would fail with only atac, this includes making a dendrogram using scipy
    if len(dataFrameList) > 1:
        ##Create the first figure, the dendrogram of ATAC-seq datasets
        distArray = ssd.squareform(matrix1)
        plt.figure(figsize=(10, 16))
        Z = scipy.cluster.hierarchy.linkage(distArray, method='single', metric='euclidean')
        dn = dendrogram(Z, labels=cellNames, orientation = "left")
        plt.savefig(outputLocation + '/cell_region_dendrogram.pdf', bbox_inches = "tight")
    else:
        print("You have only included one cell region set, this will omit the cell region dendrogram")

    print("Formatting loci:")

    ##Bring in our Loci sets
    gwasFrame = 0

    ## columns we wish to save
    final_columns = ["CHR_ID", "Start", "CHR_POS", "End", "Chromosome", "DISEASE/TRAIT"]
    if lociSelection != 0:

        if lociSelection[-3:] == "tsv":
            lociSelectDF=pd.read_csv(lociSelection,sep='\t')
        elif lociSelection[-3:] == "csv":
            lociSelectDF=pd.read_csv(lociSelection)
        tempColumnList = lociSelectDF.columns
        final_columns.extend(tempColumnList)



    ##add GWAS in tsv format
    for filename in glob.glob(gwasLocation + "/" + '*.tsv'):
        loopFrame = pd.read_csv(filename, sep="\t", low_memory=False)

        ## give a backup id by file count, in case they dont have an identity column
        s = filename[:-4]
```

```
        loopFrame["fileId"] =  (s[s.rindex('/')+1:])

        loopFrame = loopFrame[(loopFrame[loopFrame.columns[0]].notna()) & (loopFrame[loopFrame.columns[1]].notna())]

        ##These if statements are to account for various formatting mistakes
        if "DISEASE/TRAIT" not in loopFrame.columns:
            loopFrame["DISEASE/TRAIT"] = loopFrame["fileId"]
        if "Start" in loopFrame.columns:
            loopFrame["CHR_POS"] = loopFrame["Start"].astype(int)
        if "start" in loopFrame.columns:
            loopFrame["CHR_POS"] = loopFrame["start"].astype(int)
        if "Reference_name" in loopFrame.columns:
            loopFrame["Chromosome"] = loopFrame["Reference_name"]
            loopFrame["CHR_ID"] = loopFrame["Reference_name"]
        if "reference_name" in loopFrame.columns:
            loopFrame["Chromosome"] = loopFrame["reference_name"]
            loopFrame["CHR_ID"] = loopFrame["reference_name"]
        if "Chromosome" in loopFrame.columns:
            loopFrame["CHR_ID"] = loopFrame["Chromosome"]
        if "chr" in loopFrame.columns:
            loopFrame["CHR_ID"] = loopFrame["chr"]

        loopFrame["Chromosome"] = loopFrame["CHR_ID"]

        ##if gwas is missing chr part of chromosome ids than add it
        if (1 in loopFrame.Chromosome.unique()) or ('1' in loopFrame.Chromosome.unique()):
                    loopFrame["Chromosome"] = "chr" + loopFrame["Chromosome"]


        if not isinstance(gwasFrame, pd.DataFrame):
            loopFrame.drop(columns=loopFrame.columns.difference(final_columns), inplace=True)
            loopFrame = loopFrame[pd.to_numeric(loopFrame['CHR_POS'], errors='coerce').notnull()]
            loopFrame['CHR_ID'] = loopFrame['CHR_ID'].astype('category')
            gwasFrame = loopFrame
        else:
            loopFrame.drop(columns=loopFrame.columns.difference(final_columns), inplace=True)
            loopFrame = loopFrame[pd.to_numeric(loopFrame['CHR_POS'],
```

```
errors='coerce').notnull()]
            loopFrame['CHR_ID'] = loopFrame['CHR_ID'].astype('category')
            gwasFrame = pd.concat([gwasFrame, loopFrame],axis=0, ignore_index=True)


    ## add GWAS in csv format
    for filename in glob.glob(gwasLocation + "/" + '*.csv'):

        loopFrame = pd.read_csv(filename, low_memory=False)

        ## give a backup id by file count, in case they dont have an identity column
        s = filename[:-4]
        loopFrame["fileId"] =  (s[s.rindex('/')+1:])

        loopFrame = loopFrame[(loopFrame[loopFrame.columns[0]].notna()) & (loopFrame[loopFrame.columns[1]].notna())]


        ##These if statements are to account for various formatting mistakes
        if "DISEASE/TRAIT" not in loopFrame.columns:
            loopFrame["DISEASE/TRAIT"] = loopFrame["fileId"]
        if "Start" in loopFrame.columns:
            loopFrame["CHR_POS"] = loopFrame["Start"].astype(int)
        if "start" in loopFrame.columns:
            loopFrame["CHR_POS"] = loopFrame["start"].astype(int)
        if "Reference_name" in loopFrame.columns:
            loopFrame["Chromosome"] = loopFrame["Reference_name"]
            loopFrame["CHR_ID"] = loopFrame["Reference_name"]
        if "reference_name" in loopFrame.columns:
            loopFrame["Chromosome"] = loopFrame["reference_name"]
            loopFrame["CHR_ID"] = loopFrame["reference_name"]
        if "Chromosome" in loopFrame.columns:
            loopFrame["CHR_ID"] = loopFrame["Chromosome"]
        if "chr" in loopFrame.columns:
            loopFrame["CHR_ID"] = loopFrame["chr"]

        loopFrame["Chromosome"] = loopFrame["CHR_ID"]

        ##if gwas is missing chr part of chromosome ids than add it
        if (1 in loopFrame.Chromosome.unique()) or ('1' in loopFrame.Chromosome.unique()):
                    loopFrame["Chromosome"] = "chr" + loopFrame["Chromosome"]
```

```

        if not isinstance(gwasFrame, pd.DataFrame):
            loopFrame.drop(columns=loopFrame.columns.difference(final_columns), inplace=True)
            loopFrame = loopFrame[pd.to_numeric(loopFrame['CHR_POS'], errors='coerce').notnull()]
            loopFrame['CHR_ID'] = loopFrame['CHR_ID'].astype('category')
            gwasFrame = loopFrame
        else:
            loopFrame.drop(columns=loopFrame.columns.difference(final_columns), inplace=True)
            loopFrame = loopFrame[pd.to_numeric(loopFrame['CHR_POS'], errors='coerce').notnull()]
            loopFrame['CHR_ID'] = loopFrame['CHR_ID'].astype('category')
            gwasFrame = pd.concat([gwasFrame, loopFrame],axis=0, ignore_index=True)

    if not isinstance(gwasFrame, pd.DataFrame):
        sys.exit("No Loci files found")

    ##format our Loci frame by getting rid of NAN values and then getting rid of studies with non-numerical chromosome positions, if you want to fix these chromosome positions manually, you can
    print("Removing Empty Loci Values:")
    gwasNoNAN = gwasFrame[(gwasFrame["CHR_POS"].notna()) & (gwasFrame["DISEASE/TRAIT"].notna())]

    gwasNoNAN = gwasNoNAN.reset_index(drop=True)

    ## Location to save all non-numeric indexes
    nonNumericIndexes = []

    ## if chromosome position isn't an integer than check for non-int rows to remove or try to force into int
    if gwasNoNAN["CHR_POS"].dtype != np.int64:
        print("Non integer chromosome positions detected, these will be removed")
        for index, row in gwasNoNAN.iterrows():
            if isinstance(gwasNoNAN.at[index, "CHR_POS"], float):
                gwasNoNAN.at[index, "CHR_POS"] = int(gwasNoNAN.at[index, "CHR_POS"])
            elif isinstance(gwasNoNAN.at[index, "CHR_POS"], str):
                if gwasNoNAN.at[index, "CHR_POS"].isdigit():
                    gwasNoNAN.at[index, "CHR_POS"] =
```

```
int(gwasNoNAN.at[index, "CHR_POS"])
                else:
                    nonNumericIndexes.append(index)
            elif not np.issubdtype(gwasNoNAN.at[index, "CHR_POS"], int):
                nonNumericIndexes.append(index)

    ## Drop indexes of values that couldnt be forced into integer
    gwasFormatted = gwasNoNAN.drop(index=nonNumericIndexes)

    ##Create columns for turning into pyranges
    gwasFormatted["Start"] = gwasFormatted["CHR_POS"]
    if "End" not in gwasFrame.columns:
        gwasFormatted["End"] = gwasFormatted["CHR_POS"] + 1
    if "Chromosome" not in gwasFrame.columns:
        temp = '\t'.join(gwasFrame.CHR_ID.unique())
        if "chr" not in temp:
            gwasFormatted["Chromosome"] = "chr" +
gwasFormatted["CHR_ID"].astype(str)
        else:
            gwasFormatted["Chromosome"] =
gwasFormatted["CHR_ID"].astype(str)

    ##if the length is zero give it a 1 length
    gwasFormatted.loc[gwasFormatted.Start == gwasFormatted.End, "End"] =
gwasFormatted.loc[gwasFormatted.Start == gwasFormatted.End, "End"] + 1

    ## Subset by loci selection if given
    if lociSelection != 0:
        gwasNames = []

        eachLociFrame = []
        columnList = lociSelectDF.columns
        for index, row in lociSelectDF.iterrows():
            temp = lociSelectDF.iloc[[index]]
            dtName = str(list(temp[columnList[0]])[0])
            lociLoopFrame = gwasFormatted[gwasFormatted[columnList[0]] ==
list(temp[columnList[0]])[0]]
            for item in columnList:
                lociLoopFrame = lociLoopFrame[lociLoopFrame[item] ==
list(temp[item])[0]]
                dtName = dtName + str(list(temp[item])[0])
            eachLociFrame.append(lociLoopFrame)
            gwasNames.append(dtName)
            if lociLoopFrame.shape[0] == 0:
                print(dtName)
                sys.exit("One of the user given loci groups does not
```

```
exist")



    ### rename the Disease/Trait column to more easily callable DT column
    gwasFormatted = gwasFormatted.rename(columns={"DISEASE/TRAIT": "DT"})

    ## subset loci based on cutoff value
    if lociCutoff != 0:

        print("Subsetting loci based on user-defined count:")
        vc = gwasFormatted.DT.value_counts()
        highCount = list(vc[vc > int(lociCutoff)].index)
        gwasFormatted = gwasFormatted[(gwasFormatted["DT"].isin(highCount))]

    ##save pyranges for each disease/trait
    print("Turning GWAS into Pyranges Objects:")
    gwasPyranges = []
    gwasNonZeroPyranges = []
    indels = False

    if lociSelection == 0:
        gwasNames = list(set(gwasFormatted["DT"]))
        for item in set(gwasFormatted["DT"]):

            ##subset for each loci type
            loopFrame = gwasFormatted[(gwasFormatted["DT"] == item)]

            ##remove duplicate loci
            loopFrame = loopFrame.drop_duplicates(subset=['CHR_ID', 'Start', 'End'])

            ##Subset into appropriate columns and add to our list of final loci ranges
            tempRange = pr.PyRanges(loopFrame)
            loopRange = tempRange
            df = pd.DataFrame(list(zip(loopRange.Chromosome, loopRange.Start, loopRange.End)),columns =['Chromosome', 'Start', 'End'])
            df["Size"] = df["End"] - df["Start"]
            dfSingle = df[df["Size"] <= 1]
            testRange = pr.PyRanges(dfSingle)
            gwasPyranges.append(testRange)

            ##If there are indels we need to create range including all these larger than 1 bp loci, otherwise mark that it is blank
```

```
            if df.Size.max() > 1:
                print("Indels Detected in " + item + ", Statistical Method For Loci >1 bp Will Be Applied")
                indels = True
                df = df[df["Size"] > 1]
                indelLoopRange = pr.PyRanges(df)
                gwasNonZeroPyranges.append(indelLoopRange)
            else:
                gwasNonZeroPyranges.append([])

    else:
        for countIndel, item in enumerate(eachLociFrame):

            ##remove duplicate loci
            loopFrame = item.drop_duplicates(subset=['CHR_ID', 'Start', 'End'])

            ##Subset into appropriate columns and add to our list of final loci ranges
            tempRange = pr.PyRanges(loopFrame)
            loopRange = tempRange
            df = pd.DataFrame(list(zip(loopRange.Chromosome, loopRange.Start, loopRange.End)),columns =['Chromosome', 'Start', 'End'])
            df["Size"] = df["End"] - df["Start"]
            dfSingle = df[df["Size"] <= 1]
            testRange = pr.PyRanges(dfSingle)
            gwasPyranges.append(testRange)

            ##If there are indels we need to create range including all these larger than 1 bp loci, otherwise mark that it is blank
            if df.Size.max() > 1:
                print("Indels Detected in " + gwasNames[countIndel] + "Statistical Method For Loci >1 BP Will Be Applied")
                indels = True
                df = df[df["Size"] > 1]
                indelLoopRange = pr.PyRanges(df)
                gwasNonZeroPyranges.append(indelLoopRange)
            else:
                gwasNonZeroPyranges.append([])

    ##Create a reordered list of our ATAC-sets based on how they showed up in the dendrogram. This step will be skipped if there is only one ATAC-seq set
    reorderPyranges = []
    reorderDataframeList = []
    reorderCellNames = []
```

```
    if len(dataFrameList) > 1:
        ## using the leaves from our scipy Z set, resort your pyranges and dataframes to be in the correct order
        for item in reversed(leaves_list(Z)):
            reorderPyranges.append(prRanges[item])
            reorderDataframeList.append(dataFrameList[item])
            reorderCellNames.append(cellNames[item])
    else:
        reorderPyranges = prRanges
        reorderDataframeList = dataFrameList

    ## create matrices to store p-values and overlap information
    heatmapMatrix = np.zeros((len(reorderPyranges),len(gwasPyranges)))
    overlapMatrix = np.zeros((len(reorderPyranges),len(gwasPyranges)))

    ## create the chromosome ratios for every atac-seq set
    chromSize = pd.read_csv(chromosomeSize, thousands=',')
    chromSize = chromSize.sort_values(by=['Chromosome'])


    ##create weight matrix

    ##Based on window sizes divide our chromosomes into equal sized chuncks
    windowFrames = []
    for index, row in chromSize.iterrows():

        ## get the size of this particular chromsome
        chrSize = int(chromSize.at[index, "size in bp"])

        ## returned the rounded up version of the divided window size (3.01 becomes 4)
        n = math.ceil(chrSize/windowSize)

        windowSizesLoop = []
        zp = n - (chrSize % n)
        pp = chrSize//n
        for i in range(n):
            if(i>= zp):
                windowSizesLoop.append(pp + 1)
            else:
                windowSizesLoop.append(pp)

        chrWindowDF = pd.DataFrame(windowSizesLoop, columns =['Size'])
        chrWindowDF["End"] = chrWindowDF["Size"].cumsum()
        chrWindowDF["Start"] = chrWindowDF["End"] - chrWindowDF["Size"] +
```

```
1
        chrWindowDF.at[0,"Start"] = chrWindowDF.at[0,"Start"] -1
        chrWindowDF["Chromosome"] = chromSize.at[index, "Chromosome"]

        windowFrames.append(chrWindowDF)

    allWindowsDF = pd.concat(windowFrames)

    ## find overlaps and probability for each ATAC + GWAS
    listOverlaps = []
    overlapAllColumns = []

    windowsRange = pr.PyRanges(allWindowsDF)
    windowStarts = list(allWindowsDF.Start)
    windowEnds = list(allWindowsDF.End)
    windowChrs = list(allWindowsDF.Chromosome)
    dictWindows = {"Start":windowStarts, "End":windowEnds, "Size":list(allWindowsDF.Size), "Chromosome":windowChrs}

    print("Calculating Overlaps: ")
    if indels == True:
        print("Note: Indels signifigantly effect runtime")
    for count, item in enumerate(reorderPyranges):

        if( count != len(reorderPyranges) - 1):
            print(reorderCellNames[count], end = " - ")
        else:
            print(reorderCellNames[count], end = "\n")

        tempRange = windowsRange.coverage(item)
        dictWindows[reorderCellNames[count]] = list(tempRange.FractionOverlaps)

        ##if there are indels then save the number of atac peaks per each window, this will be need for calculations at the end
        if indels == True:
            dictWindows[reorderCellNames[count] + "PeakCount"] = list(tempRange.NumberOverlaps)

        ##find the number of overlaps
        for count2, item2 in enumerate(gwasPyranges):
            tempRange2 = windowsRange.coverage(item2)
            dictWindows[reorderCellNames[count] + gwasNames[count2]] = list(tempRange2.NumberOverlaps)

            ## If Indels exist we need to do some more expansive
```

```
calculations
            if indels == True:

                ## if indels exist in this particular gwas set continue on to indel caluclations
                if gwasNonZeroPyranges[count2]:

                    ##Grab all indels that overlap with the current ATAC set
                    indelRangeTemp = gwasNonZeroPyranges[count2].coverage(item)
                    indelOverlap = indelRangeTemp.df

                    ## keep only those that overlap
                    indelOverlap = indelOverlap[indelOverlap["NumberOverlaps"] > 0]

                    ##now create a list of every bp where an indel exists at
                    indelOverlap = indelOverlap.reset_index(drop=True)
                    indelOverlap['range']=indelOverlap.apply(lambda x : list(range(x['Start'],x['End'])),1)
                    indelChroDict = {}
                    for item in list(set(indelOverlap.Chromosome)):
                        indelChroLoop = indelOverlap[indelOverlap["Chromosome"] == item]
                        indelLoopLocations = [item for sublist in indelChroLoop["range"] for item in sublist]
                        indelChroDict[item] = indelLoopLocations

                    indelExtraOverlap = []

                    ##Get the number of these overlaps for each window so we can add them to the count
                    indelDoubleOverlap = indelOverlap.drop(['range', 'NumberOverlaps'], axis=1)
                    indelDoubleOverlap = pr.PyRanges(indelDoubleOverlap)
                    doubleOverlapCount = windowsRange.coverage(indelDoubleOverlap)
                    doubleOverlapList = list(doubleOverlapCount.NumberOverlaps)

                    ## for each window space, find how much length of indel exists in the window
                    for windowCount, windowItem in enumerate(windowStarts):
```

```
                        if windowChrs[windowCount] in indelChroDict:
                            indelWindowSize = [num for num in indelChroDict[windowChrs[windowCount]] if num <= windowEnds[windowCount] and num >= windowStarts[windowCount]]
                        else:
                            indelWindowSize = []

                        ## if none exist, add zero to list of indel overlap, if it does add the total number of bp that do
                        if len(indelWindowSize) == 0:
                            indelExtraOverlap.append(0)
                        else:
                            indelSize = len(indelWindowSize)
                            indelExtraOverlap.append(indelSize)

                    ##grab the number of peaks per window
                    listCount = dictWindows[reorderCellNames[count] + "PeakCount"]

                    ##multiply the extra length we need to add to every atac peak within every window
                    listSize = [a*b for a,b in zip(listCount,indelExtraOverlap)]

                    ## take this extra size to calculate an added probability
                    listProb = [a/b for a,b in zip(listSize, dictWindows["Size"])]

                    ##Add this probability to the base probability for a gwas specific probability
                    dictWindows[reorderCellNames[count]+ gwasNames[count2] + "Prob"] = [a+b for a,b in zip(dictWindows[reorderCellNames[count]], listProb)]

                    ##Add number of indel overlaps
                    dictWindows[reorderCellNames[count] + gwasNames[count2] + "IndelOverlap"] =  doubleOverlapList

                    ## Get the total number of indels in region to add to count
                    totalIndelRange = windowsRange.coverage(gwasNonZeroPyranges[count2])
                    totalIndelList = list(totalIndelRange.NumberOverlaps)
```

```
                    ## add the count of indels to the original gwas count
                    dictWindows[reorderCellNames[count] + gwasNames[count2]] = [a+b for a,b in zip(dictWindows[reorderCellNames[count] + gwasNames[count2]], totalIndelList)]


    ## check to see if you can make a subfolder
    if not os.path.exists(outputLocation+ '/overlaps'):
        os.mkdir(outputLocation + '/overlaps')
        if not os.path.exists(outputLocation + '/overlaps'):
            print("Output folder cannot be modified, so overlaps will not be saved. Try running again with higher permissions.")
            overlapSave = False
        else:
            overlapSave = True
    else:
        overlapSave = True

    print("Calculating P-Values: ")
    for count, item in enumerate(reorderPyranges):

        if( count != len(reorderPyranges) - 1):
            print(reorderCellNames[count], end = " - ")
        else:
            print(reorderCellNames[count], end = "\n")

        for count2, item2 in enumerate(gwasPyranges):
            overlapCoverage = item.coverage(item2)
            if overlapSave:
                overlapCoverageFrame = overlapCoverage.df
                overlapCoverageFrame = overlapCoverageFrame[overlapCoverageFrame["NumberOverlaps"] != 0]
                overlapCoverageFrame.to_csv(outputLocation + '/overlaps' + '/overlap_' + gwasNames[count2][:10] + '_' + reorderCellNames[count][:10] + '.txt',sep='\t')
            overlapValue = sum(list(item.coverage(item2).NumberOverlaps))
            overlapMatrix[count,count2] = overlapValue
            if indels == True:
                overlapValue = overlapValue + sum(dictWindows[reorderCellNames[count] + gwasNames[count2] + "IndelOverlap"])


            ## if there are not indels, use the normal probabilities, if there are indels, use the gwas specific window probabilites
```

```
            if indels == False:
                out1, out2 = zip(*filter(all, zip(list(dictWindows[reorderCellNames[count] + gwasNames[count2]]), list(dictWindows[reorderCellNames[count]]))))
            elif gwasNonZeroPyranges[count2]:
                out1, out2 = zip(*filter(all, zip(list(dictWindows[reorderCellNames[count] + gwasNames[count2]]), list(dictWindows[reorderCellNames[count]+ gwasNames[count2] + "Prob"]))))
            else:
                out1, out2 = zip(*filter(all, zip(list(dictWindows[reorderCellNames[count] + gwasNames[count2]]), list(dictWindows[reorderCellNames[count]]))))

            ## since psinib is a statistical method, sometimes root function finds results well outside the region near 0, this check accomodates this by turning all values over 1 into 1
            valueSinib = psinib(overlapValue-1, out1, out2, lowerTail=False)
            if valueSinib <= 1:
                heatmapMatrix[count,count2] = valueSinib
            else:
                print("P-Value equal greater than one found. note: Sinib sometimes uses a statistical approximation of a p-value for non convergent cases, this can allow for impossible p-values. These  values are set to 1 in results.")
                heatmapMatrix[count,count2] = 1
    try:
        print("Lowest P-Value: " + str(heatmapMatrix.min()))
    except ValueError:
        pass
    try:
        if heatmapMatrix.max() > 1:
            print("P-Value greater than one found in final matrix.")
    except ValueError:
        pass


    newHeatMatrix = heatmapMatrix
    ax = sns.heatmap(newHeatMatrix, cmap="YlGnBu",annot=True,xticklabels=gwasNames)
    ax.xaxis.set_ticks_position('top')
    ax.xaxis.set_label_position('top')
    plt.xticks(rotation=90)
    plt.savefig(outputLocation + '/pvalues_heatmap.pdf', bbox_inches = "tight")

```

```
    ## Create the final figure
    figure, axis = plt.subplots(2, 2, figsize=(40, 30))
    sns.heatmap(newHeatMatrix, cmap="YlGnBu", cbar=False, ax=axis[1, 1],xticklabels=gwasNames)
    axis[1, 1].xaxis.set_ticks_position('top')
    axis[1, 1].xaxis.set_label_position('top')
    plt.xticks(rotation=90)
    sns.heatmap(newHeatMatrix, cmap="YlGnBu", annot=True, ax=axis[0, 0])

    axis[0, 1].grid(False)
    axis[0, 1].axis('off')
    if len(dataFrameList) > 1:
        dendrogram(Z,  labels=cellNames, orientation = "left", ax=axis[1, 0])
    else:
        axis[1, 0].text(0.1, 0.5, cellNames[0], horizontalalignment='right', verticalalignment='center', transform=axis[1, 0].transAxes)
        axis[1, 0].grid(False)
        axis[1, 0].axis('off')
    axis[1, 1].margins(x=0)
    plt.savefig(outputLocation + '/complete_figure.pdf', bbox_inches = "tight")

    ##save the matrix of p-values in case they want to do something else with the formatting
    newHeatFrame = pd.DataFrame(newHeatMatrix)
    newHeatFrame.columns = gwasNames
    if len(dataFrameList) > 1:
        newHeatFrame.index = reorderCellNames
    else:
        newHeatFrame.index = cellNames

    newHeatFrame.to_csv(outputLocation + '/pvalue_matrix.txt',sep='\t')


    ##save overlap Counts
    newOverlapMatrix = overlapMatrix

    ## save the matrix of overlaps count in case they want to do something else with the formatting
    newOverlapFrame = pd.DataFrame(newOverlapMatrix)
    newOverlapFrame.columns = gwasNames
    if len(dataFrameList) > 1:
        newOverlapFrame.index = reorderCellNames
    else:
```

```python
        newOverlapFrame.index = cellNames

    ## save the matrix of overlap counts in case this information is desired
    newOverlapFrame.to_csv(outputLocation + '/overlap_count_matrix.txt',sep='\t')



## Sinib Package Functions
## translated from R to Python from: https://github.com/boxiangliu/sinib

def q(u,p):
    return([(i * math.exp(u)) / (1 - i + i * math.exp(u)) for i in p])

def K(u,n,p):
    return(sum([a*math.log(1-b+b*math.exp(u)) for a,b in zip(n,p)]))

def Kp(u,n,p):
    return(sum([a*b for a,b in zip(n,q(u,p))]))

def Kpp(u,n,p):
    q2=q(u,p)
    return(sum([a*b*(1-b) for a,b in zip(n,q2)]))

def Kppp(u,n,p):
    q2=q(u,p)
    return(sum([a*b*(1-b)*(1-2*b) for a,b in zip(n,q2)]))

def Kpppp(u,n,p):
    q2=q(u,p)
    return(sum([a*b*(1-b)*(1-6*b*(1-b)) for a,b in zip(n,q2)]))

def saddlepoint(u,n,p,s):
    return(Kp(u,n,p)-s)

def w(u,n,p):
    K2=K(u,n,p)
    Kp2=Kp(u,n,p)
    return(np.sign(u)*math.sqrt(2*u*Kp2-2*K2))

def u1(u,n,p):
    Kpp2=Kpp(u,n,p)
    return((1-math.exp(-u))*math.sqrt(Kpp2))

def p3(u,n,p,s,mu):
```

```
    w2=w(u,n,p)
    u12=u1(u,n,p)
    if ((u12==0.0) or (s==mu)):
        Kpp0=sum([a*b*(1-b) for a,b in zip(n,p)])
        Kppp0=sum([a*b*(1-b)*(1-2*b) for a,b in zip(n,p)])
        return(1/2-(2*np.pi)**(-1/2)*((1/6)*Kppp0*Kpp0**(-3/2)-(1/2)*Kpp0**(-1/2)))

    else:
        return(1-norm.cdf(w2)-norm.pdf(w2)*(1/w2-1/u12))


def u2(u,n,p):
    return(u*math.sqrt(Kpp(u,n,p)))

def k3(u,n,p):
    return(Kppp(u,n,p)*Kpp(u,n,p)**(-3/2))

def k4(u,n,p):
    return(Kpppp(u,n,p)*Kpp(u,n,p)**(-2))

def p4(u,n,p,s,mu):

    u2b=u2(u,n,p)
    k3b=k3(u,n,p)
    k4b=k4(u,n,p)
    wb=w(u,n,p)
    p3b=p3(u,n,p,s,mu)
    u1b=u1(u,n,p)
    if ((u1b==0) or (s==mu)):
        return(p3b)
    else:
        return(p3b-norm.pdf(wb)*((1/u2b)*((1/8)*k4b-(5/24)*k3b**2)-1/(u2b**3)-k3b/(2*u2b**2)+1/wb**3))

def calc_p4(s, n, p, mu):
    if (s == sum(n)):
        p4b=numpy.prod([a**b for a,b in zip(p,n)])
    else:
        test = root(lambda x: saddlepoint(x,n,p,s), 0)
        u_hat = test.x[0]
        if not test.success:
            n_trial=100000
            n_binom=len(p)
```

```
            mat = np.random.binomial(n, p, (n_trial, n_binom)).T

            S = np.sum(mat,axis=0)
            counterTemp = 0
            s = [s]*len(S)
            for l1,l2 in zip(S,s):
                if l1 >= l2:
                    counterTemp = counterTemp + 1
            p4_ = counterTemp/len(S)
        else:
            p4_=p4(u_hat,n,p,s,mu)

    return(p4_)

def psinib(q,size,prob,lowerTail=True,logP=False):

    if(not all(isinstance(x, int) for x in size)):
        sys.exit('Non-integer values were used for size values given to psinib')
    if not all(isinstance(x, int) for x in size):
        n = [int(i) for i in size]
    else:
        n = [int(i) for i in size]

    p = [float(i) for i in prob]
    s = int(q)

    mu = sum([a*b for a,b in zip(n,p)])

    p4b = calc_p4(s+1,n,p, mu)

    if(lowerTail):
        if (logP):
            return(np.log(1-p4b))
        else:
            return(1-p4b)

    else:
        if(logP):
            return(np.log(p4b))
        else:
            return(p4b)

## Main Function which parses arguments and returns values

def main():
```

```

    ## parse the given arguments to see if the neccesary libraries have been given and
    parser = argparse.ArgumentParser(prog ='gaia',
                                     description ='Compare ATAC-seq data to loci.')

    parser.add_argument('-a', '--cellRegion', required = True,
                        help='the folder location of the cell region bed files stored in .txt format')
    parser.add_argument('-g', '--loci', required = True,
                        help='the folder location of the loci files stored in .tsv format')
    parser.add_argument('-c', '--chrom', required = True,
                        help='the location of the chromsome size file stored in a .csv format')
    parser.add_argument('-o', '--output', required = True,
                        help='the folder location you want the results to be output into')
    parser.add_argument('-u', '--peakUniqueness',  default=0, required = False, type=int,
                        help='a cutoff value for ATAC uniqueness (e.g. if given 12, then any atac peak found in more than 12 atac sets will be removed from all of them) - by default uniqueness is not considered')
    parser.add_argument('-l', '--lociCutoff',default=0, required = False, type=int,
                        help='a loci cutoff value, will only consider loci groups (phenotypes or cohorts) with more loci than this cutoff value - by default this cutoff is 0')
    parser.add_argument('-s', '--specificLoci',default=0, required = False,
                        help='a txt file with the specific loci phenotype you would like to use. This can be very helpful if using a large loci set with with many phenotypes, and you want to sort by more than just loci count.' )
    parser.add_argument('-m', '--maskRegion',default=0, required = False,
                        help='a txt file containing a set of regions that you want to subset each ATAC region by, for example a set of regions around the TSSs of genes of interest. This will reduce the ATAC regions to just those that overlap with this set of regions' )

    parser.add_argument('-w', '--windowSize',  default=100000, required = False, type=int,
                        help='Size of the window you would like to create around each loci to make the statistical comparison for enrichment')

```

```
    args = parser.parse_args()

    gaiaAssociation(args.cellRegion, args.loci, args.chrom, args.output, args.peakUniqueness, args.lociCutoff, args.specificLoci, args.maskRegion, args.windowSize)

```
