# Supplementary material for: Regulatory landscape enrichment analysis (RLEA): a computational toolkit for non-coding variant enrichment and cell type prioritization
Source: BMC Bioinformatics. 2024 May 7;25:179. doi: 10.1186/s12859-024-05794-7 (PMC11075237; doi:10.1186/s12859-024-05794-7)
Supplement: Supplementary file 3 — Additional file 3: Supplementary Figure 1. GaiaAssociation reveals. De novo variant enrichment in regulatory regions of brain structures and glutamatergic neurons in individuals with epilepsy. De novo variants in individuals with epilepsy taken from the denovo-db database were analyzed using GaiaAssociation for cell type prioritization. The analysis consisted of 433 DNVs tested against 44 cell type regulatory profiles. GaiaAssociation highlighted four brain structures and one neuronal cell type. The orange dashed line at −log10 (P) = 1.30 represents a p-value cutoff of ≤ 0.05. These results highlight the applicability of GaiaAssociation to de novo variant data. [file 12859_2024_5794_MOESM3_ESM.pdf]

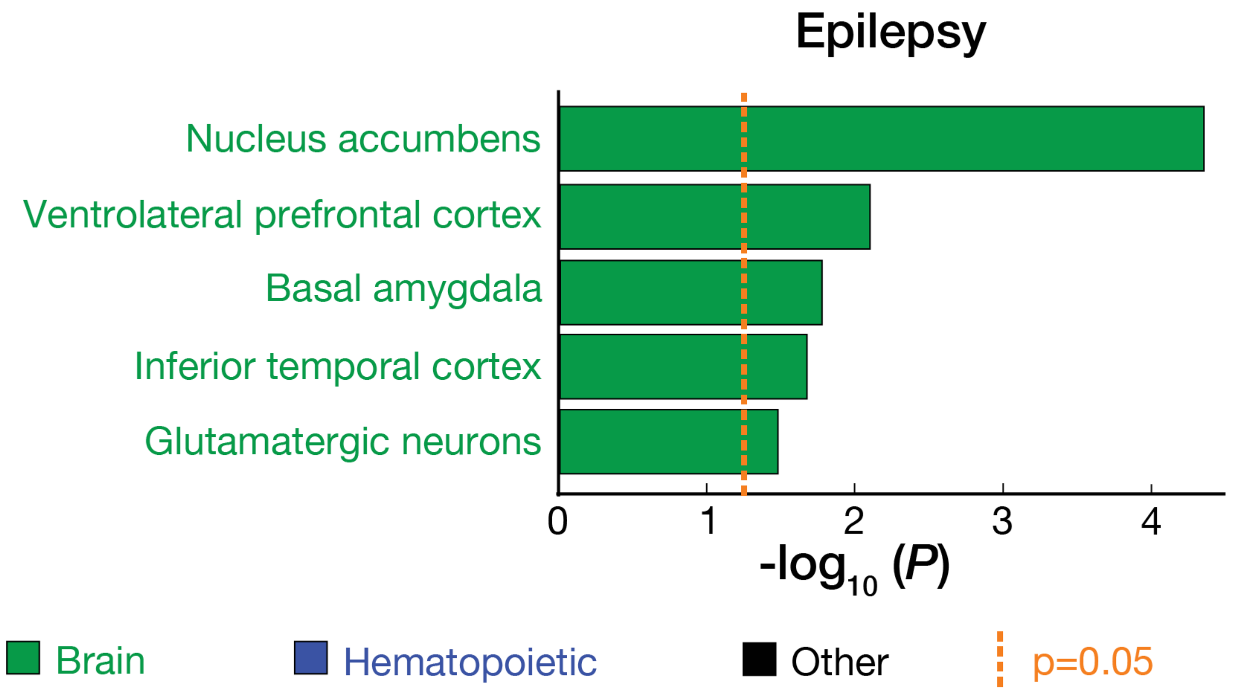

Supplementary Figure 1: GaiaAssociation reveals *de novo* variant enrichment in regulatory regions of brain structures and glutamatergic neurons in individuals with epilepsy. D*e novo* variants in individuals with epilepsy taken from the denovo-db database were analyzed using GaiaAssociation for cell type prioritization. The analysis consisted of 433 DNVs tested against 44 cell type regulatory profiles. GaiaAssociation highlighted four brain structures and one neuronal cell type. The orange dashed line at $-\log_{10}(P) = 1.30$ represents a p- value cutoff of ≤ 0.05. These results highlight the applicability of GaiaAssociation to *de novo* variant data.
