# Supplementary material for: Regulatory landscape enrichment analysis (RLEA): a computational toolkit for non-coding variant enrichment and cell type prioritization
Source: BMC Bioinformatics. 2024 May 7;25:179. doi: 10.1186/s12859-024-05794-7 (PMC11075237; doi:10.1186/s12859-024-05794-7)
Supplement: Supplementary file 4 — Additional file 4: Supplementary Figure 2. Analysis of the true positive rate (TPR) in GaiaAssociation across increasing SNP and enrichment levels. The TPR was tested using randomly genotyped and imputed SNPs from the UK Biobank database. These SNPs were overlapped against ATACseq data from glutamatergic neurons. Analyses were performed across seven GaiaAssociation window sizes. We studied a naïve GaiaAssociation window (entire genome) and GaiaAssociation windows ranging from to 25,000 – 1,000,000 bp. Each power analysis was conducted across enrichments of (a) 30%, (b) 35%, (c) 40%, (d) 45%, and (e) 50%. Enrichments are defined by the proportion of SNPs that overlap ATAC-seq regions (compared to those that do not); specifically the percentage over the expected value of the naive binomial distribution in the absence of any association. When the number of SNPs and enrichment are both low, the naive window size has an advantage as it does not account for the unsimulated association between SNPs and ATAC-seq peaks, explaining its higher FPR observed in Fig. 3a. At moderate to higher values of enrichment and SNP numbers, the power of RLEA and the naive binomial are equivalent, with RLEA having the advantage of a lower FPR. The TPR rate is achieved by all window sizes at lower SNP counts, and by all methods equivalently at higher enrichments [file 12859_2024_5794_MOESM4_ESM.pdf]

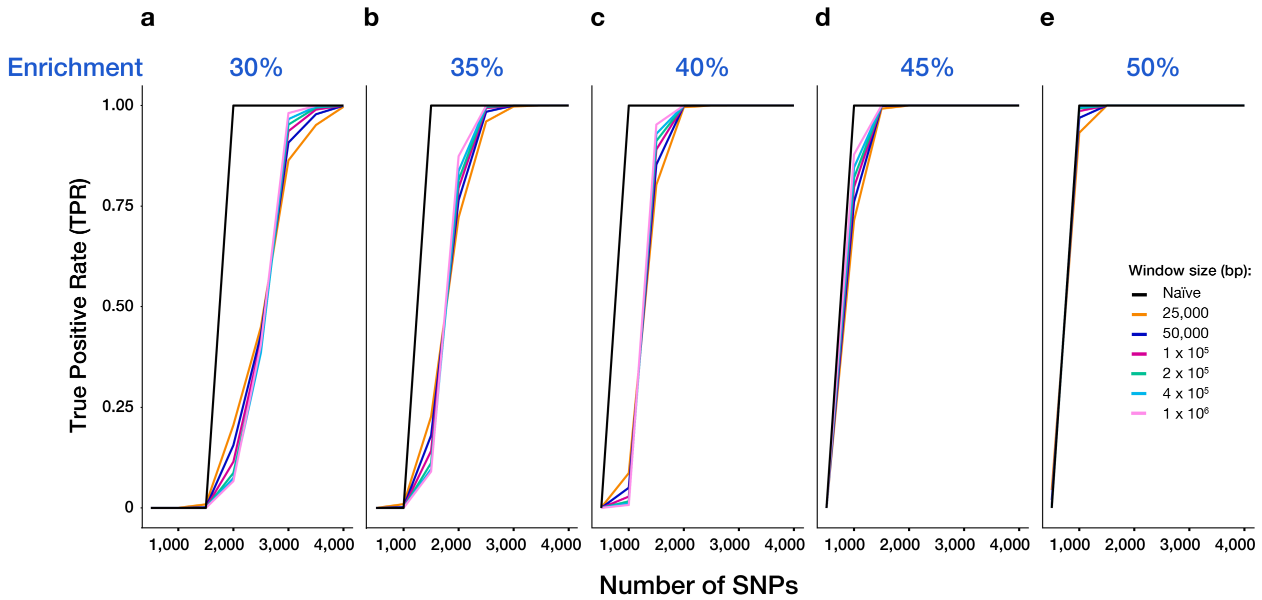

Supplementary Figure 2: Analysis of the true positive rate (TPR) in GaiaAssociation across increasing SNP and enrichment levels. The TPR was tested using randomly genotyped and imputed SNPs from the UK Biobank database. These SNPs were overlapped against ATAC-seq data from glutamatergic neurons. Analyses were performed across seven GaiaAssociation window sizes. We studied a naïve GaiaAssociation window (entire genome) and GaiaAssociation windows ranging from to 25,000 – 1,000,000 bp. Each power analysis was conducted across enrichments of (**a**) 30%, (**b**) 35%, (**c**) 40%, (**d**) 45%, and (**e**) 50%. Enrichments are defined by the proportion of SNPs that overlap ATAC-seq regions (compared to those that do not); specifically the percentage over the expected value of the naive binomial distribution in the absence of any association. When the number of SNPs and enrichment are both low, the naive window size has an advantage as it does not account for the unsimulated association between SNPs and ATAC-seq peaks, explaining its higher FPR observed in **Figure 3a**. At moderate to higher values of enrichment and SNP numbers, the power of RLEA and the naive binomial are equivalent, with RLEA having the advantage of a lower FPR. The TPR rate is achieved by all window sizes at lower SNP counts, and by all methods equivalently at higher enrichments.
