# Supplementary material for: Regulatory landscape enrichment analysis (RLEA): a computational toolkit for non-coding variant enrichment and cell type prioritization
Source: BMC Bioinformatics. 2024 May 7;25:179. doi: 10.1186/s12859-024-05794-7 (PMC11075237; doi:10.1186/s12859-024-05794-7)
Supplement: Supplementary file 5 — Additional file 5. Supplementary Figure 3: Cell type prioritization by GaiaAssociation analysis is unaffected by the GWAS SNP p-value threshold. Cell type prioritization by GaiaAssociation was compared between GWAS variants from the EMBL-EBI GWAS catalog possessing a p-value ≤ 5.0 x 10^−8^ (x-axis) and ≤ 1.0 x 10^−5^ (y-axis) across 44 cell type regulatory regions. Studies highlighted in Figure 2b (labelled a-f) and three additional studies from Figure 2a – systemic lupus erythematosus (g), leukocyte count (h), and inflammatory bowel disease (i) – were used for comparative analysis. Cell type prioritization between variants from both p-value groups demonstrated strong concordance and positive association (R2 ranges: 0.82 - 0.996) providing evidence that SNP selection by p-value threshold does not influence the selection of cell type. Cell types colored in red received an RLEA p-value ≤ 0.001 and achieved multiple testing significance with variants from both p-value thresholds. Cell types colored in pink and green received a RLEA p-value of ≤ 0.05 and > 0.05, respectively. The linear diagonal trendline (dashed red line) indicates that the 44 cell types were ranked analogously between both variant groups. Notably, the most highly ranked cell type (labelled in each plot) was concordant between both p-value groups across all nine GWAS studies. [file 12859_2024_5794_MOESM5_ESM.pdf]

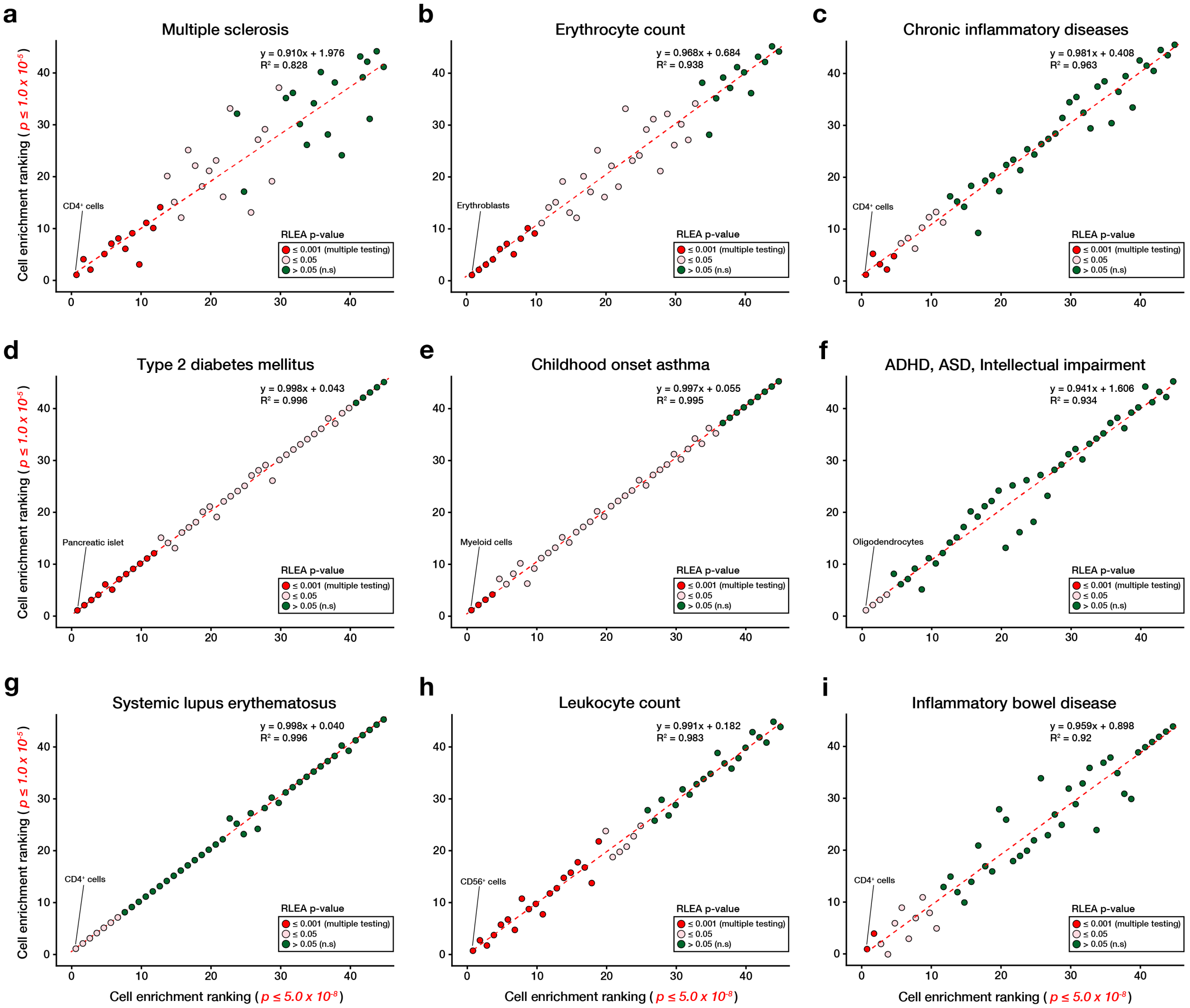

Supplementary Figure 3: **Cell type prioritization by GaiaAssociation analysis is unaffected by the GWAS SNP p-value threshold.** Cell type prioritization by GaiaAssociation was compared between GWAS variants from the EMBL-EBI GWAS catalog possessing a p-value ≤ 5.0 x 10^{-8} (x-axis) and ≤ 1.0 x 10^{-5} (y-axis) across 44 cell type regulatory regions. Studies highlighted in **Figure 2b** (labelled **a-f**) and three additional studies from **Figure 2a** – systemic lupus erythematosus (**g**), leukocyte count (**h**), and inflammatory bowel disease (**i**) – were used for comparative analysis. Cell type prioritization between variants from both p-value groups demonstrated strong concordance and positive association (R^2 ranges: 0.82 - 0.996) providing evidence that SNP selection by p-value threshold does not influence the selection of cell type.

Cell types colored in red received an RLEA p-value ≤ 0.001 and achieved multiple testing significance with variants from both p-value thresholds. Cell types colored in pink and green received a RLEA p-value of ≤ 0.05 and > 0.05, respectively. The linear diagonal trendline (dashed red line) indicates that the 44 cell types were ranked analogously between both variant groups. Notably, the most highly ranked cell type (labelled in each plot) was concordant between both p-value groups across all nine GWAS studies.
