# Supplementary material for: Regulatory landscape enrichment analysis (RLEA): a computational toolkit for non-coding variant enrichment and cell type prioritization
Source: BMC Bioinformatics. 2024 May 7;25:179. doi: 10.1186/s12859-024-05794-7 (PMC11075237; doi:10.1186/s12859-024-05794-7)
Supplement: Supplementary file 6 — Additional file 6: Supplementary Figure 4. Comparative enrichment analysis between RLEA by GaiaAssociation and GREGOR. GaiaAssociation performance was compared against GREGOR using ATAC-seq regions from 44 cell types and the GWAS studies highlighted in Fig. 2b. While there was a general concordance of enrichment for cell type rankings between the methods, GREGOR tended to show a non-specific enrichment in significance across most or all cell types, whereas GaiaAssociation was generally more selective, especially for the ADHD, ASD, and Intellectual impairment results in (a) and the schizophrenia results in (b). [file 12859_2024_5794_MOESM6_ESM.pdf]

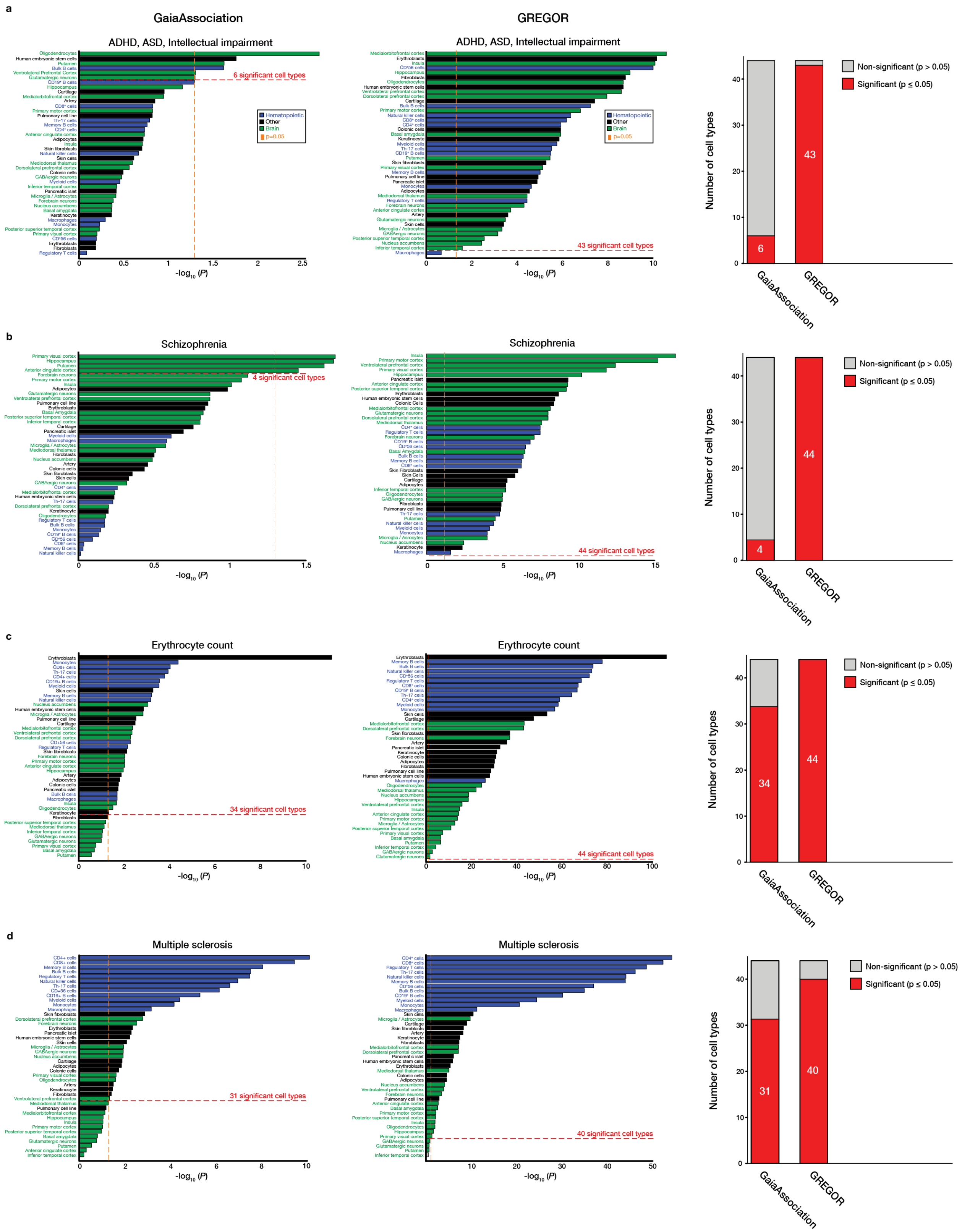

Supplementary Figure 4: Comparative enrichment analysis between RLEA by GaiaAssociation and GREGOR. GaiaAssociation performance was compared against

GREGOR using ATAC-seq regions from 44 cell types and the GWAS studies highlighted in **Figure 2b**. While there was a general concordance of enrichment for cell type rankings between the methods, GREGOR tended to show a non-specific enrichment in significance across most or all cell types, whereas GaiaAssociation was generally more selective, especially for the ADHD, ASD, and Intellectual impairment results in (**a**) and the schizophrenia results in (**b**).
