# Supplementary material for: Regulatory landscape enrichment analysis (RLEA): a computational toolkit for non-coding variant enrichment and cell type prioritization
Source: BMC Bioinformatics. 2024 May 7;25:179. doi: 10.1186/s12859-024-05794-7 (PMC11075237; doi:10.1186/s12859-024-05794-7)
Supplement: Supplementary file 8 — Additional file 8: Supplementary Methods 1. Utilizing GREGOR and SpecVar for the purposes of comparisons with GaiaAssociation. [file 12859_2024_5794_MOESM8_ESM.pdf]

Supplementary Methods 1. Utilizing GREGOR and SpecVar for the purposes of comparisons with GaiaAssociation.

GREGOR

https://genome.sph.umich.edu/wiki/GREGOR

To run GREGOR in the comparative analysis against GaiaAssociation, we followed the provided GREGOR documentation, with modifications as necessary. The steps were as follows:

- GREGOR is based on the perl language, so Perl must be downloaded prior to using it. For Mac users, Perl should already be installed for OS > 10. You can confirm whether Perl is installed by typing “perl -help” into terminal or command line. If you do not see the Perl documentation, then either Perl is not installed, or your PATH needs to be modified so you can use Perl on the command line.
- Once Perl is installed, you must install GREGOR as outlined in the documentation, this will create a folder for GREGOR where analysis MUST be performed.
- GREGOR is built on a set of pre-assembled reference libraries. Since these reference libraries (split into AFR, AMR, ASN, EUR, SAN) are based on different LD variables, they are split into different libraries based on different R^2 values. You should select a reference population and an R^2 value which you believe best matches the interests of your question, in this instance we used the EUR population with $R^2 > 0.7$.
- Once your chosen reference is downloaded, it must be unpacked as described in the GREGOR documentation.
- To run GREGOR, a config file is used to define the parameters and locations of all relevant files. An example config file is provided within GREGOR which should be modified by the user to define whether the task should be run in a batch managed mode, the relevant LD variables, as well as the location of the OCR bed files and SNP files.

SpecVar

https://github.com/AMSSwanglab/SpecVar

To facilitate replicating the comparison we performed between SpecVar and GaiaAssociation, we outline the general process of installing, setting up, and finding relevant GWAS data for SpecVar here. This process will be highly dependent on both the software and hardware setup of the user, so we have tried to provide general problem-solving suggestions where relevant.

To run SpecVar in the comparative analysis performed against GaiaAssociation, we followed the provided SpecVar documentation, with modifications as was necessary. The steps were as follows:

- Specvar is built on LDSC and HOMER, so each must be installed based on their own documentation provided in hyperlinks. You must then notate the bin locations of each in the SpecVar_RT.sh file.

- Specvar requires both a Python2 and Python3 environment simultaneously, both of which must be manually notated as bins in the same SpecVar_RT.sh file, as above. Since certain managers, such as anaconda, do not support Python2 instances for some newer CPUs, this may require research into the particular situation and manager you wish to use. We followed this guide to set up a Python 2.7 environment on a Mac M1, Sonoma 14.3.1.
- Homer must be added to the bash PATH variable as well. Adding to the bash path is different between various OS, so attempt to follow the guide in HOMER's documentation, otherwise a cursory search on Google for "your OS + modify bash path variable" should help.
- For any GWAS data, LDSC requires alignment to hg19 (GRCh37), the EAF, effective sample size, variant & effect allele, and the variant's RSID, many of which may not be available in publicly available summary statistics, older GWAS studies, or non-standardized GWAS summary statistic repositories. Since SpecVar is built on LDSC it has the same requirements. Ensure your data matches the requirements given in the SpecVar GitHub documentation.
- SpecVar can only find mediating contexts for a single GWAS set at a time, so this process must be repeated for each study, and each study set will have to be given a unique name for the .bed and .txt files.
